# mPPases create a conserved anionic membrane fingerprint as identified via multi-scale simulations

**DOI:** 10.1371/journal.pcbi.1010578

**Published:** 2022-10-03

**Authors:** Alexandra O. M. Holmes, Adrian Goldman, Antreas C. Kalli

**Affiliations:** 1 School of Biomedical Sciences and Astbury Centre for Structural Molecular Biology, University of Leeds, Leeds, United Kingdom; 2 Research Program in Molecular and Integrative Biosciences, University of Helsinki, Helsinki, Finland; 3 Leeds Institute of Cardiovascular and Metabolic Medicine and Astbury Centre for Structural Biology, University of Leeds, Leeds, United Kingdom; University of Virginia, UNITED STATES

## Abstract

Membrane-integral pyrophosphatases (mPPases) are membrane-bound enzymes responsible for hydrolysing inorganic pyrophosphate and translocating a cation across the membrane. Their function is essential for the infectivity of clinically relevant protozoan parasites and plant maturation. Recent developments have indicated that their mechanism is more complicated than previously thought and that the membrane environment may be important for their function. In this work, we use multiscale molecular dynamics simulations to demonstrate for the first time that mPPases form specific anionic lipid interactions at 4 sites at the distal and interfacial regions of the protein. These interactions are conserved in simulations of the mPPases from *Thermotoga maritima*, *Vigna radiata* and *Clostridium leptum* and characterised by interactions with positive residues on helices 1, 2, 3 and 4 for the distal site, or 9, 10, 13 and 14 for the interfacial site. Due to the importance of these helices in protein stability and function, these lipid interactions may play a crucial role in the mPPase mechanism and enable future structural and functional studies.

## Author summary

In this work, we demonstrated conservation of lipid-interaction sites on proteins from two species that deviated from their evolutionary common ancestors a long time ago, using the membrane-integral pyrophosphatases from a thermophilic bacteria species and a plant species. We further showed that these sites are preserved in homology modelled proteins of the same family. This retention of a common lipid interaction profile or “fingerprint” and our ability to predict this for other proteins in this family may indicate that they are more integral to protein function than previously thought. By identifying lipid interactions that may act to stabilise the protein structure, these properties could be exploited to solve protein structures, and the interfacial site’s potential involvement in inter-subunit communication may be useful for further investigation of the catalytic cycle of this clinically relevant membrane protein family.

Word Count: 140

## Introduction

Membrane integral pyrophosphatases (mPPases) are a family of membrane proteins responsible for coupling the hydrolysis of the pyrophosphate (PP_i_) phosphoanhydride bond to the pumping of a cation across the membrane [1]. This allows mPPases to both remove excess PP_i_ from the cytoplasm, and to generate a membrane potential. mPPases are found in all kingdoms of life, excluding fungi and multicellular animals [2]. Due to this, they are validated selectively toxic drug targets against a variety of protozoan and bacterial pathogens [3–6] and reducing the function of mPPases *via* novel inhibitors may play a role in combatting these infectious pathogens.

**Fig 1:**
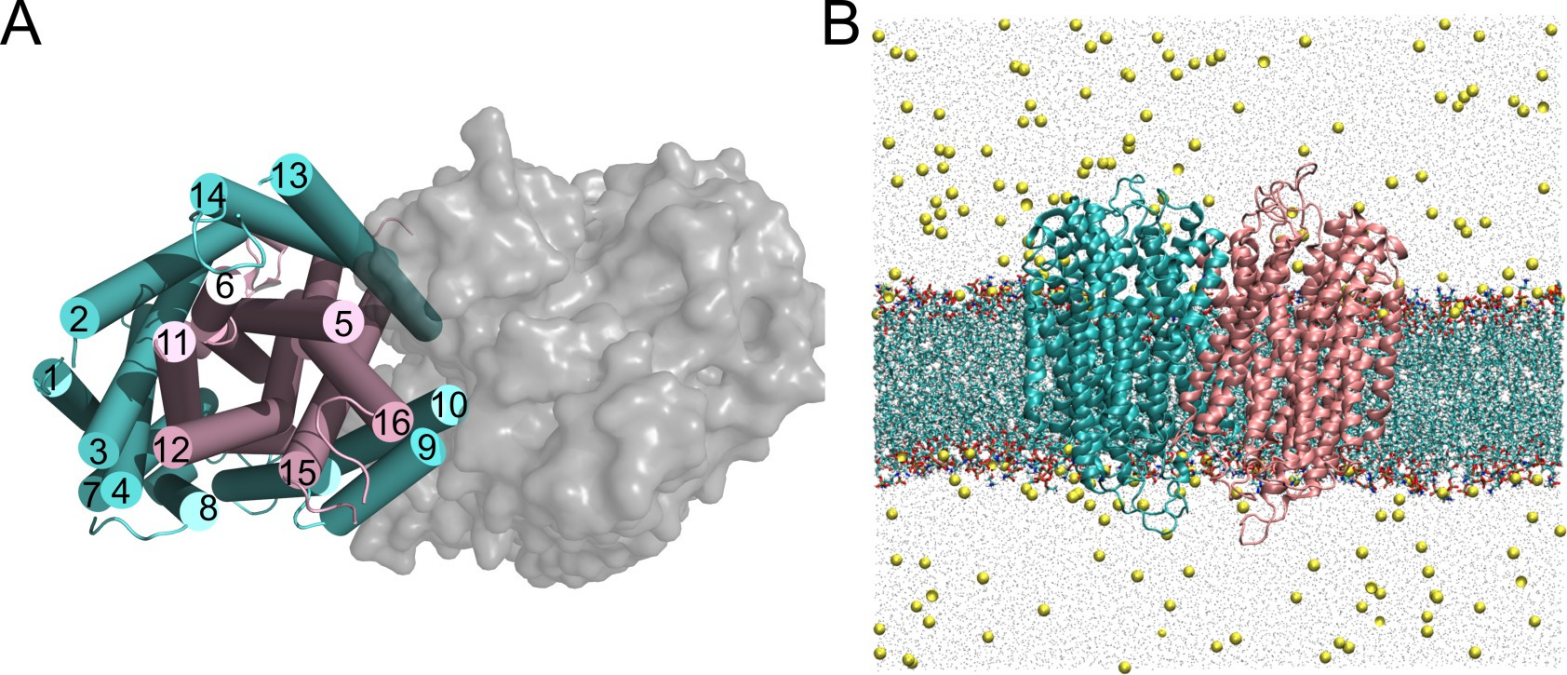
The structure of *Tm*-PPase and simulation box set up. A) the *Tm*-PPase resting state crystal structure (PDB: 4AV3) viewed from above to demonstrate the concentric circles of TMH of each subunit (outer ring in cyan and inner in pink) and the TMH contributing to the interface with the adjoining subunit (grey surface representation). B) The atomistic simulation box where the protein subunits are each coloured in cyan and pink and shown as Cartoon representation, the POPE (80%) and POPA (20%) bilayer is shown as lines representation and the water particles are grey points, and the ions (NaCl 150 mM) are shown as yellow VDW particles.

Crystal structures revealed that mPPases exist as homodimers, where each subunit is composed of 16 transmembrane helices (TMH) [7–11]. A single subunit is formed by two concentric rings of TMH: the inner ring (TMH 5, 6, 11, 12, 15 and 16) makes up the 4 catalytic regions: the catalytic centre, the coupling funnel, the ionic gate and the exit channel, while the outer ring (TMH 1, 2, 3, 4, 7, 8, 9, 10, 13, 14) forms the subunit-subunit interface and the membrane-facing surface of the protein (**Fig 1A**). Despite their common structure, seven different mPPase subfamilies have been functionally characterised [12–14]. In short, mPPase catalytic activity is either K^+^ dependent or K^+^ independent and the pumping specificity is either H^+^ only, Na^+^ only, dual H^+^/Na^+^ or H^+^ regulated by Na^+^. Of these subfamilies, only the 3D structures of the K^+^-dependent H^+^-PPase from *Vigna radiata* (*Vr-*PPase) [7], and the K^+^-dependent Na^+^-PPase from *Thermotoga maritima* (*Tm-*PPase) [8–10] have been resolved to high resolution.

These 3D structures have facilitated understanding of the mechanism by which mPPases perform hydrolysis and ion pumping. However, there is conflict surrounding the order of these two events [15,16] and more recently, data have been published indicating that the mechanism is more complicated than previously thought [10,17]. There is also a dispute around how the mPPase subunits operate with one another, which may be explained by the environment of the protein: lipidated [17] or detergent-solubilised [10], and so could indicate protein-lipid interactions [1]. Despite evidence that the lipid environment may have a role in regulating the function of mPPases, the interaction of mPPases with their lipid environment is still unknown.

In recent years, molecular dynamics (MD) simulations have played a role in uncovering and studying membrane protein-lipid interactions [18]. These interactions can have multiple effects on the protein of interest, for example modulating stability [19], assisting conformational changes [20], oligomerisation [21,22] and large-scale protein organisation [23]. MD simulations represents a robust way to identify putative lipid binding sites which can be refined through further simulations or in tandem with other methods [18].

In this study, we examined the interactions and dynamics of three mPPases structures in model lipid bilayers *via* multi-scale MD simulations. Our results suggest that mPPases form an anionic annulus in the membrane and possess specific anionic lipid binding sites at the dimer interface and the distal regions of the protein. These protein-lipid interactions are conserved between mPPases from different species with differing pumping specificities, which may suggest that these are a general property of mPPases.

## Results

### *Tm-*PPase forms an anionic lipid fingerprint in the membrane

**Fig 2:**
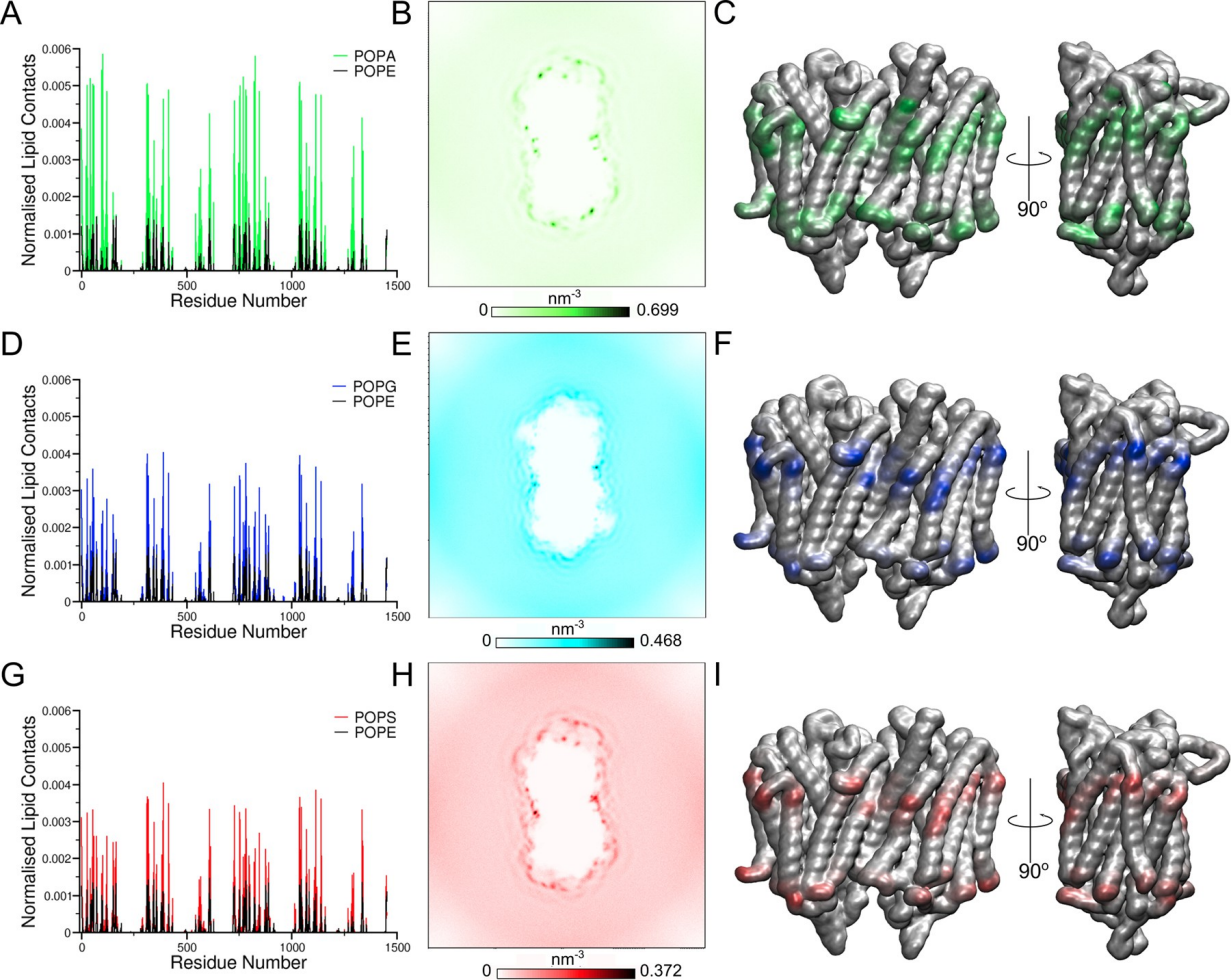
*Tm*-PPase interacts preferentially with anionic lipids at four distinct sites. The contacts between the lipid bilayer and *Tm-*PPase for model bilayers consisting of 80% POPE and 20% anionic lipid represented by A) normalised lipid contacts with both POPE and POPA/G/S and the residue number of the protein, B) the density of the PO4 bead of the CG anionic lipid around the protein, where the trajectory has been fitted to the protein backbone and C) the contacts of the PO4 atom represented on the 3D structure of the protein with a colour scale from white to green, for POPA, D-F) blue for POPG, and G-I) red for POPS. The lipid contacts were normalised by dividing the number of contacts of each residue with the length of simulation and the number of lipid species.

We first performed simulations with the 3D structure of the *Tm-*PPase, due to the extensive structural characterisation of the protein over the last decade [8–10]. The *Tm-*PPase structure was inserted into bilayers containing POPE and POPA, POPG or POPS molecules (Table 1) (**Fig 1B**). Little is known about the specific compositions of the *T*. *maritima* native lipid bilayers [24–26] and so palmitoyl-oleoyl phospholipids were considered an appropriate proxy. Following 5 μs of simulation, the normalised contacts of the protein with the anionic component in the inner leaflet of the bilayer were higher for all residues compared with the other lipid components, suggesting that the anionic lipids POPA, POPG and POPS interacted preferentially with *Tm*-PPase in the cytoplasmic leaflet compared to the zwitterionic POPE lipids (**Fig 2)**. Titration of the concentration of each anionic lipid, (40%, 20% and 30% (made up of 10% of each anionic lipid) of the overall lipid concentration) demonstrated that this annulus was retained in all concentrations with ~20 lipids interacting with the protein in each concentration (**S1 Fig**). Therefore, the 20% anionic lipid bilayers were chosen for further experiments, so selective interactions could be distinguished.

**Table 1:** Details of the lipids used in the paper.

Lipid	Class	Abbreviation	Tails	Charge
Cholesterol	Sterol	-	C(d18:1/18:0)	0
Ceramide hexoside	Sphingolipid	DPCE	C(d18:1/18:0)	0
Phosphatidylethanolamine	1-Palmitoyl-2-Oleoyl	POPE	C16:0/18:1	0
Phosphatidylcholine		POPC		0
Phosphatidylglycerol		POPG		-1
Phosphatidic acid		POPA		-2
Phosphatidylserine		POPS		-1
Phosphatidylinositol bisphosphate	Phosphatidylinositol	PIP_2_	C16:1(9c), C18:1(9c)	-5

**Table 2:** Simulation details.

Protein	Name	% Anionic Lipid	Mutations*	CG simulations	AT simulations
*Tm-*PPase	TmPPE100	0	-	5 x 5 (μs)	-
	TmMix10	10 each^a^	-	5 x 5 (μs)	-
	TmPA20 TmPG20 TmPS20	20	-	5 x 5 (μs)	3 x 250 (ns)
	TmPA20_SSM TmPG20_SSM TmPS20_SSM		K^9.70^A K^10.49^A K^13.52^A K^14.48^A^b^	5 x 5 (μs)	-
	TmPA20_DSM TmPG20_DSM TMPS20_DSM		K^9.70^A K^10.49^A K^13.52^A K^14.48^A	5 x 5 (μs)	3 x 250 (ns)
	TmPA40 TmPG40 TmPS40	40	-	5 x 5 (μs)	-
*Vr-*PPase	VrTonoplast	12 cumulative	-	4 x 5 (μs) 1 x 20 (μs)	3 x 250 (ns)
	VrTonoplast_DSM		K^10.49^A K^13.48^A K^14.40^A K^14.48^A	5 x 5 (μs)	3 x 250 ns
*Cp-*PPase	CpMix10	10 each^a^	-	5 x 5 (μs)	-
	CpPA20 CpPG20 CpPS20	20	-	5 x 5 (μs)	3 x 250 (ns)
	CpPA20_DSM CpPG20_DSM CpPS20_DSM		K^9.69^A K^9.73^A R^13.52^A R^14.48^A	5 x 5 (μs)	3 x 250 (ns)

^a^here the membrane was composed of equal numbers POPA, POPG and POPS

^b^these mutations were to a single interfacial interaction site

*Ballesteros and Weinstein numbering system is used

Analysis of the interactions between the *Tm*-PPase and the anionic lipids allowed us to identify lipid binding sites through contact analysis and lipid density. These binding sites were identified using the top 5% of interacting residues from the contact analysis. This analysis revealed four symmetrical anionic lipid binding sites at the dimer interface and the “distal regions” of the protein (**Fig 2B and 2C**). Nine positive lysine and arginine residues (R^1.60^ (27), K^1.61^ (28), R^2.38^ (43), K^3.59^ (96), R^3.63^ (100), R^3.67^ (104), K^4.40^ (120), K^8.40^ (311) and K^8.41^ (312)) [note that the Ballesteros and Weinstein numbering system is used [13,27] alongside traditional residue numbering] formed each of the distal interaction sites. The residues involved in the dimer interface sites were four positive lysine residues (K^9.70^ (389), K^10.49^ (415), K^13.52^ (568) and K^14.48^ (609)), two of which were located in one subunit and two in the other subunit.

Analysis of the lipid contacts revealed that POPA interacted more frequently with *Tm*-PPase compared to POPG and POPS at 20% anionic lipid bilayer content (**Fig 2D–2I**). To examine further the more frequent binding of POPA lipids, we also performed a simulation in which the bilayer contained an equal anionic lipid mix (10% each of POPA, POPG and POPS). In this simulation, POPA interacted up to 43-fold or 84-fold more frequently than POPG or POPS, respectively, supporting our previous observation that POPA interacts more with *Tm*-PPase compared to other anionic lipids (**S1D Fig)**. Therefore, further characterisation of the *Tm-*PPase lipid binding sites in this work was performed using the system that contained *Tm*-PPase with 20% POPA lipids in the bilayer (TmPA20 in Table 2) system, but it was found that, for all of the parameters assessed, the interactions with POPG and POPS were reduced compared to those with POPA.

Contact analysis and PyLipID [28] were used to understand the lipid occupancy, exchange and residence of the distal and interfacial sites. The distal site was able to accommodate more lipids, with 2 lipids bound the majority of the time, rarely increasing to 3 or in some cases 4 for short periods (**S1V–S1W Fig**). Residues R^2.38^ (43), R^3.63^ (100) and K^8.40^ (311) had the highest lipid residence times (1.41 μs, 0.23 μs and 0.13 μs, respectively) (**S4 Fig**) and were found to primarily coordinate 2 or 3 lipids in the site, with the 4^th^ lipid briefly interacting with other residues in the site. The interfacial site was able to accommodate fewer lipids at any time, usually being occupied by one or occasionally two lipids. Rarely a third lipid would interact with the site, but this was with only one of the outer residues (typically K^14.48^ (609)), rather than being coordinated within the site. This is consistent with the size of the sites, as the interfacial site is much smaller than the distal site, and is situated in an interfacial cleft, causing the inner residues to be less accessible for contacts. Additionally, the rate of lipid exchange was higher in the interfacial site than the distal site, with residence times averaging 0.28 μs for the four interfacial residues (**S4 Fig**).

### *Vr-*PPase in its native bilayer forms a similar membrane fingerprint

To examine whether the anionic lipid fingerprint identified above was *Tm-*PPase specific or if it also occurs in mPPases in other species and in other membranes, we also performed simulations using the crystal structure of *Vr-*PPase. Like *Tm-*PPase, *Vr-*PPase is structurally well-characterised but mesophilic rather than thermophilic and, as there are no resting state *Vr-*PPase structures available, the relaxed product bound state (PDB: 5GPJ) was chosen for simulation to be the most comparable to the *Tm-*PPase findings.

**Fig 3:**
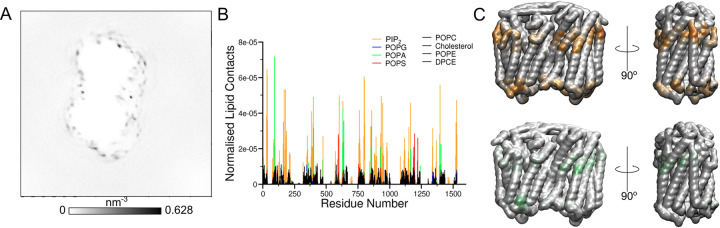
*Vr*-PPase in its native bilayer forms an anionic membrane fingerprint. The interactions between *Vr-*PPase and a realistic tonoplast membrane represented through A) the average density of the phosphate particles of the anionic lipids (PIP_2_, POPG, POPS and POPA) calculated using a combined trajectory fitted around the protein, B) the normalised number of contacts between the lipids in the bilayer and the residues of the protein, alongside C) a representation of the contacts on the 3D protein structure of PIP_2_ (orange) and POPA (green).

As we have more information about the *V*. *radiata* tonoplast membrane, simulations of *Vr-*PPase were performed in bilayers resembling this membrane (29% cholesterol, 25% POPC, 17% POPE, 17% ceramide hexoside, 6% PIP_2_, 3% POPG, 2% POPS and 1% POPA) [29]. The four anionic interaction sites seen with *Tm-*PPase were present at the interfacial and distal regions of the *Vr-*PPase protein (**Fig 3A**), where lysine and arginine residues formed the distal site (K^1.60^ (35), K^1.62^ (37), K^1.67^ (42), K^2.50^ (94), K^3.59^ (162), R^4.33^ (177), K^4.37^ (181), R^4.44^ (188), K^8.41^ (352), K^8.52^ (363)). However, unlike *Tm-*PPase, the distribution of residues forming the interfacial site was uneven, as one from one subunit and three from the other contributed to forming interactions with the anionic lipids (K^10.49^ (457), K^13.48^ (595), K^14.40^ (632), K^14.48^ (640) for *Vr-*PPase). These simulations were run with 323 K temperature as in our previous simulations, but protein-lipid interactions were very similar to the same simulation in which the temperature was 310 K (**S7 Fig**).

As these simulations contained additional anionic lipids compared to the systems with *Tm*-PPase, we were able to study the preference of *Vr*-PPase for anionic lipids in a more realistic membrane. All of the anionic lipids formed interactions with the interfacial and distal interaction sites, but our analysis suggests that interactions between *Vr*-PPase and PIP_2_ were highly favoured over the neutral and other anionic lipids (**Fig 3B**). Of the palmitoyl-oleoyl phospholipids, POPA was the favoured lipid over both POPG and POPS, despite its low representation in the membrane (1%) (**S2A–S2H Fig**). Therefore, the interactions with PIP_2_ and POPA were used to understand the properties of the *Vr-*PPase sites (**Fig 3C**).

Despite having similar structural arrangements of the interfacial and distal sites as in *Tm-*PPase, the interactions between the lipids and the protein were somewhat different in *Vr-*PPase. The interfacial site appears to be smaller and only accommodates one anionic lipid at a time for the majority of the 20 μs of simulation time, with a second lipid interacting with the binding site very rarely. However, when there are two lipids interacting with the binding side, one lipid interacts with residues K^13.48^ (595) and K^14.48^ (632) that are at the centre of this binding site and the second lipid interacts with one of the outer residues of the site (K^10.49^ (457) and K^14.40^ (640)) (**S2Q–S2R Fig**). Despite this, the average residence time in the interfacial site was longer than that seen for *Tm-*PPase, at 0.51 μs (**S5 Fig**). The residence time analysis at each protein residue revealed that there was a lot of variability in the residence times of the different residues in the binding site, and residue K^4.37^ (181) (1.25 μs residence time) had the most prolonged interactions with lipids in this site compared to the others (**S5 Fig**). The distal interaction site also appeared to be smaller for *Vr-*PPase compared to *Tm-*PPase, as it formed contacts with a maximum of 3 lipids, but primarily 1 or 2. The reduced occupancy may be due to the increased size of the PIP_2_ head group and availability of negative charge to form interactions with, thereby reducing the availability of binding site residues or the tilting of TMH 13 and 14 towards the interface compared compressing the site compared to *Tm-*PPase in the resting state [9].

### Homology-modelled crystallography target *Cp-*PPase retains this fingerprint

Our results above suggest the two structurally characterised mPPases induce an anionic lipid fingerprint in model membranes. As other mPPase studies have also indicated that lipid binding could be of interest for functional and stabilising studies [17,30], we modelled the Na^+^/H^+^-PPase from *C*. *leptum* (*Cp-*PPase; see Methods for details) to examine whether the anionic lipid fingerprint is found in other members of this family. Despite producing two high quality models from both Robetta and AlphaFold2 with significant similarity (1.05 Å RMSD), the Robetta homology model (reference PDB ID: 5GPJ) was chosen for this study as the arrangement of the distal helices was more in keeping with those seen in *Tm-*PPase and *Vr-*PPase structures (**S10 Fig**).

*Clostridium* species are predicted to contain POPG, POPE, POPS and cardiolipin, in addition to diradylglycerols. For this reason, we simulated this structure in the same bilayers as *Tm*-PPase. This also enables better comparison of our results between the two systems. The *Cp*-PPase simulations were run with 20% POPA, POPG or POPS or an equal anionic lipid mix (10% each). As well as being a crystallographic target, *Cp*-PPase is also a different subtype of mPPase compared to *Tm-*PPase and *Vr-*PPase; this allowed us to examine whether the anionic interactions identified for two different mPPases are retained in other subfamilies.

As in the other systems, *Cp-*PPase formed an anionic lipid fingerprint in the membrane (**S3 Fig**), where specific interactions formed between the lipids and positively charged residues at the distal site (R^1.56^ (23), K^1.60^ (27), K^2.46^ (51), R^2.47^ (52), K^3.59^ (101), R^7.62^ (290), K^7.64^ (292)) and at the interfacial site (K^9.69^ (361), K^9.73^ (365), R^13.52^ (543), R^14.48^ (584)). The interfacial site was made up of two residues from each protein subunit and *Cp-*PPase displayed a preference for POPA over the anionic lipids POPG or POPS in the mixed simulations. This supports our previous hypothesis that binding of anionic lipids to these two types of interaction site may be a general property for all mPPases.

Interestingly, the distal and interfacial sites of *Cp-*PPase better resembled that of *Tm-*PPase, despite being modelled with reference to the *Vr-*PPase structure, both in structural arrangement and lipid occupancy and average residence time. The interfacial site accommodates up to 2 anionic lipids at a time and the distal site could accommodate up to 4 (**S6 Fig**), despite being comprised of only 7 positive residues, similar to the interactions seen with *Tm-*PPase in the same bilayer systems. However, the sites have shorter average residence times than in the other mPPase simulations, with only 0.1 μs for the distal site, primarily contributed to by K^2.46^ (51), R^2.47^ (52), K^3.59^ (101), R^7.62^ (290), and the interfacial site had an average residence time of 0.2 μs.

### *In silico* mutations disrupt the protein-lipid interactions

**Fig 4:**
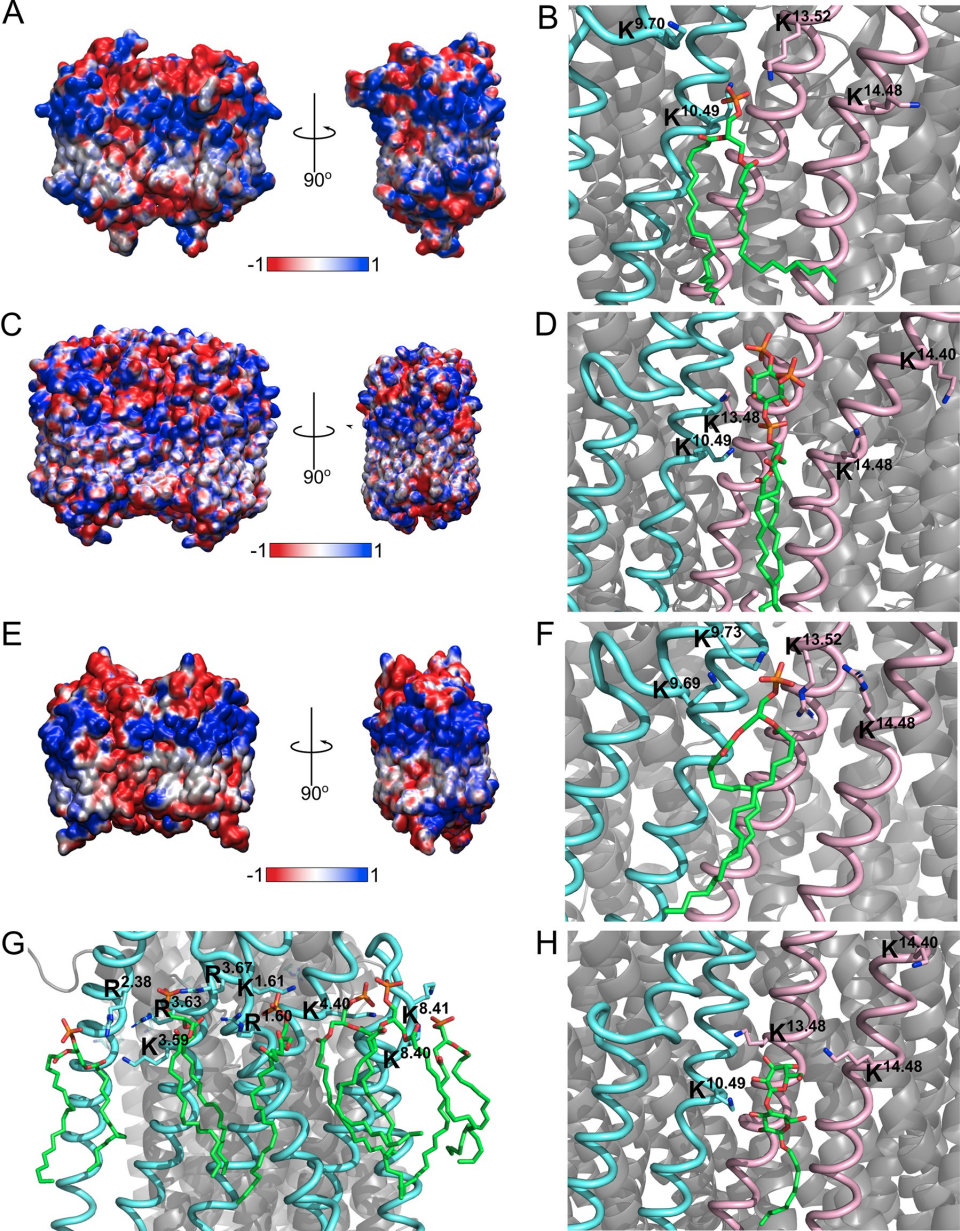
Electrostatic profiles and arrangements of the mPPases lipid binding sites. The electrostatic profile as calculated by APBS and structural arrangement of the A-B) *Tm-*PPase, C-D) *Vr-*PPase and E-F) *Cp-*PPase protein interfacial anionic lipid binding site with POPA (green sticks) or PIP_2_ in the case of *Vr*-PPase bound and the interacting lysines or arginines shown as sticks. The snapshots are the final frame of one of the CG simulations (at 5 μs of simulation) converted to an atomistic representation. G) The distal interaction site of *Tm-*PPase, and H) the arrangement of a DM detergent molecule (green sticks) in the crystal structure of *Vr-*PPase (PDB: 4A01).

To examine whether there was any synergy in the binding of lipids in the lipid binding sites identified above, we performed *in-silico* mutagenesis of the four positively charged residues in the interfacial site to alanine. Analysis of the lipid density following identical simulation to the wild-type (WT) proteins showed that when a single or double site mutation (DSM) was made, the anionic lipid binding was lost, but binding at the intact site remained. This, therefore, indicates that our simulations do not demonstrate any cooperativity between the binding sites. This confirmed that the anionic lipid binding at these sites was due to specific interactions with the protein, rather than a stochastic effect, and that the binding is independent at each site. To assess whether this was due to the properties of the protein surface in these areas, electrostatic analysis of the proteins was performed and revealed that positively charged regions matched the location of the anionic binding at the interfacial and distal regions of the protein (**Fig 4**). The electrostatic profiles were very similar between the three mPPases in this study, as well as the arrangement of their binding site residues (**Fig 4**).

### Refinement of the lipid interactions *via* atomistic simulations

The effect of lipid binding on the dimeric arrangement or stability of the protein could not be assessed through these coarse-grained (CG) simulations, as the elastic network between the protein monomers maintained the protein-protein interface and conformational state, while allowing flexible regions to move (**S8 Fig**). Therefore, to refine the protein-lipid interactions at these sites and identify any effect the lipid binding had on protein structure and dynamics, the final coordinates of the WT or DSM *Tm-*PPase, *Vr*-PPase and *Cp-*PPase systems were converted to an AT representation (see Methods). Frames were chosen where at least one anionic lipid was bound to all of the anionic lipid interaction sites so that their binding could be better understood at an atomistic resolution.

The association of the anionic lipids at the WT protein interface was maintained throughout the 250 ns simulations, as was predicted with the CG residence times, and interactions with all the proposed lysine and arginine residues at the mPPase protein interface and distal interaction sites were retained. Analysis of the simulations revealed interacting residues that had not been predicted by the initial CG simulations, such as with Y^9.64^ (383), Y^13.45^ (561) and Y^13.51^ (567) for *Tm*-PPase (**S13 Fig**). However, these appear to only interact a proportionately small amount of the time compared to the lysine and arginine residues. In all these simulations there appeared to be no differences between the two interfacial binding sites in each mPPase or in the dynamics between the mutated and WT proteins throughout the atomistic simulations (**S8 and S9 Figs**). However, the RMSD of the proteins in the systems TmPA20_DSM, TmPG20_DSM and CpPS20 did not appear to flatten throughout the 250 ns simulations, which may be due to the high flexibility of the 5–6 and 13–14 loops, as seen in the RMSF analysis (**S8 Fig)**.

## Discussion

In this study, we have created atomistic models of three different mPPases in modelled membranes. Our work with *Vr*-PPase represents a realistic model of an mPPase in a complex bilayer that mimics its native environment. Our simulations have shown that mPPases inserted into a bilayer create an anionic lipid fingerprint around them with preferential interactions with anionic lipids at two distinct sites. In mPPases, two pertinent studies of catalytic asymmetry and inter-subunit communication reported different changes in PP_i_ affinity at the second monomer following binding of substrate at the first. One of the major differences in these experiments was whether the mPPases were in a lipid [17] or detergent [10] environment. These data suggest that lipid binding could have a modulatory role in inter-subunit communication and catalytic asymmetry, and delipidation through solubilisation and purification procedures thereby influences mPPase function.

In this investigation, mPPases were found to interact preferentially with anionic lipids compared to neutral lipids. This interaction appears to be a result of the charged surface of the protein, as demonstrated by the electrostatic profile analysis and loss of interaction following mutation and loss of charge. In addition to this, the pronounced preference for POPA in *Tm-*PPase may be due to its increased negative charge compared to the POPG and POPS lipids (-2 vs -1) or due to its similarities with the *T*. *maritima* native dietherglycerol phospholipids lipids, as both are anionic and do not possess headgroups [26]. This may also be the case for the *Cp*-PPase preferential interactions with POPA. Non-specific lipid interactions have been proposed to affect protein function. For example, cardiolipin binding to UraA (H^+^-uracil symporter) [31], cytochrome *bc*_1_ (respiratory chain complex) [32] and SecY (proton translocon) [33] is thought to attract positive ions for pumping. It is unclear whether this functionality exists for other anionic lipids, as it is believed that this proton trap capability comes from the presence of two phosphate groups with different p*K*_a_ values [34]. However, more recent studies suggest the p*K*_a_ values are similar for each cardiolipin phosphate [35,36], so potentially this functionality is applicable to the single-phosphate anionic lipids in this investigation. Therefore, the anionic lipid clustering around the mPPases could serve to attract the pumped cations and the Mg_2_PP_i_ for catalysis.

The observed anionic interactions were primarily localised to the cytoplasmic leaflet (**Figs 2 and S12**). Charged regions at the cytoplasmic side of proteins were shown to have a role in membrane protein insertion into the bilayer [37]. However, the clustering of anionic lipids to these areas and the specific interactions identified in this study may also play role in the function and stability of mPPases. Similar interactions between the lysine and arginine residues of the identified binding sites and the anionic lipid headgroups were also found in other proteins [38–40], in which the lipid interactions had marked effect on protein stability, function or dynamics. Additionally, it was more recently demonstrated for several oligomeric membrane-integral transporters that interfacial lipids play a crucial role in oligomerisation and stability [21,22]. Lipids are also considered capable of stabilising transient conformational states [20]. The need to stabilise alternative states of the catalytic cycle and different members of the mPPase family for structural studies means that MD-identified lipid interactions may be a key part of achieving structural information, particularly as interfacial binding sites were identified in this study.

Our studies identified two kinds of lipid binding site on mPPases, an interfacial binding site and a distal binding site. The residues at the interfacial study are not highly conserved, ranging from 47.8%– 61.8% conservation. This is low for functionally relevant residues, but there are the repeated interactions with residues around K^9.7X^, such as K^9.70^ (*Tm-*PPase) and K^9.73^ (*Cp-*PPase), around K/R^13.52^ (K in *Tm-*PPase and *Vr-*PPase (K^13.48^) and R in *Cp-*PPase) and residues around K/R^14.48^ (K^14.48^ in *Tm-*PPase, K^14.48^ in *Vr-*PPase and R^14.48^ in *Cp-*PPase). These differ in position by one helix turn, and so face out into the membrane, thereby preserving their function and potentially accommodating the change in headgroup size between POPA and PIP_2_. In addition to conservation of interactions with specific residues, they are positioned on functionally relevant helices. This clustering of interactions on TMH that form the dimer interface and are linked to inter-subunit communication and K^+^-dependency clearly supports our hypothesis that presence of anionic lipids at the interfacial interaction site may be functionally relevant to the mPPase catalytic cycle.

In addition to the computational evidence laid out in this work, there is experimental evidence of binding at the interfacial site. In the highest resolution mPPase structure currently available (*Vr*-PPase (PDB: 4A01)) [7], n-decyl-β-D-maltopyranoside (DM) detergent molecules are situated in this proposed binding site. In particular, the binding of one of these is highly similar to the anionic lipid positioning seen in our CG and AT simulations. Moreover, hydrogen bonds form between this detergent molecule and the K^9.70^ and K^10.49^ residues, mirroring the *Tm-*PPase simulations. The structural evidence of binding at the interfacial site in a mPPase provides further support to this region acting as a lipid binding site. The replacement of lipid with detergent was likely due to the purification and crystallisation conditions promoting removal of even tightly bound lipids [41]. However, the binding of this detergent may have acted similarly to a lipid and helped maintain oligomeric stability, particularly as it has been suggested that detergents can bind in lipid interaction sites [41].

The interacting residues at the distal interaction site are not highly conserved (20.8%– 64%), but interactions cluster at similar structural positions. In all proteins in this study, interactions between anionic lipids and the proteins were formed with TMH 1, 2, 3 and 4, with R/K^1.60^ (R in *Tm-*PPase, and K in *Vr-*PPase *Cp-*PPase). Positively charged residues within one helix turn from R/K^1.60^, at K^1.61^ (*Tm-*PPase), K^1.62^ (*Vr-*PPase) and R^1.56^ (*Cp-*PPase) and residues in the midpoints of the helices: K^3.59^, R^3.63^ and R^4.48^ (*Tm-*PPase), K^2.50^, K^3.59^ and K^4.44^ (*Vr*-PPase), and K^2.46^, R^2.47^ and K^3.59^ (*Cp-*PPase) also formed large number of interactions with anionic lipids. In previous simulations of *Tm-*PPase, the distal region of the protein has been found to be flexible [42], and does not have high conservation between species. Therefore, our simulations suggest that the interactions are conserved to specific helix turns rather than individual residues. This might explain why despite the lower sequence similarity, we observe similar interactions with anionic lipids in the distal area. The distal lipid interaction sites may play a role in mPPase stability, as the region was identified during a case study of the IMPROvER (integral membrane protein stability selector) pipeline for stabilising mutations [43]. Further, three of the *Cp-*PPase residues identified by IMPROvER were found to interact with anionic lipids in our study (F^1.53^, R^3.67^ and R^7.62^). We were unable to study effects of lipid interaction on protein stability, as in the CG simulations the elastic force network prevents the protein structure from substantially changing, although the loops were able to move. Conversion to all-atom systems partly overcame this, as the elastic force network was removed. Despite this, due to the corroborative results between this study and the IMPROvER study, the distal lipid interaction sites found in this work may play a role in protein stability.

Our study also provides evidence that lipid interactions in the distal and interfacial region may be a more general property of mPPase subfamilies, as *Tm-*PPase is a K^+^-dependent Na^+^-PPase, *Vr-*PPase is a K^+^-dependent H^+^-PPase and *Cp-*PPase is a K^+^-dependent Na^+^/H^+^-PPase. Despite the conserved pattern of interactions in our studies, there was no conserved sequence motif to identify these interactions in other family members, so likely homology modelling and electrostatic profile analysis, as performed here for *Cp-*PPase, would be required to identify binding sites in other homologues. The retention of interactions between pumping specificities is perhaps not unexpected, as the residues involved in coordinating the pumped ion are at the centre of the mPPase structure [1]. However, the conservation across subfamilies of lipid interactions that may stabilise the protein and be of mechanistic relevance bodes well for future structural and functional research using alternative homologues.

The preferential interaction of *Vr-*PPase with PIP_2_ in this work was striking, as PIP lipids are known to have roles in signalling and protein-protein interactions and are often found bound to proteins of interest [44]. The function of *Vr-*PPase in the tonoplast membrane of plants has been linked to auxin regulation and signalling [45] and cooperative function with soluble pyrophosphatases [46]; this, taken with the evidence of PIP_2_ binding in this study, may suggest a mechanism by which this signalling is able to take place. Additionally, the roles of the vacuolar ATPase complex and *Vr-*PPase are closely aligned [1], which may also be mediated by PIP_2_ binding and activity.

This work provides the first evidence that interactions can form between mPPases and anionic lipids and that these are in regions that may hold functional significance and are conserved across homologues. These observations are very promising for future mPPase research. The role of the distal interaction site in stability needs to be investigated further, but co-crystallisation with lipids or mutagenesis to stabilise this region in detergent may be promising for structural studies and characterisation of other mPPases. Additionally, the putative role of the interfacial site in inter-subunit communication and catalysis warrants further investigation as it may be another factor in the apparent increasing complexity of the mPPase catalytic cycle.

## Methods

### Structure preparation and modelling

The coordinates of the resting-state crystal structures of *Tm*-PPase at 2.6 Å (PDB: 4AV3) [8] and *Vr-*PPase at 3.5 Å (PDB: 5GPJ) [9] were prepared for simulation by the addition of unresolved solvent-facing loop regions (residues 1, 30, 211–221, and 577–595 in *Tm-*PPase and 1–3, 39–62, and 262–278 in *Vr-*PPase) or mutations using Modeller [47].

We submitted the protein sequence of the mPPase from *Clostridium leptum* (*Cp-*PPase) sequence to the Robetta (robetta.bakerlab.org) server [48] and AlphaFold2 [49] for homology modelling. Both of these programmes produced models of *Cp-*PPase similar to crystallographic mPPase structures. Model quality analysis was performed using SWISS-MODEL Tools (swissmodel.expasy.org/qmean) for QMEAN (qualitive model energy analysis) and Z-score analysis using the QMEANBrane option [50], the results of which indicated that these models were of sufficient quality for simulation in this study (**S10 Fig**). The Robetta model was chosen for simulation as the arrangement of helices in this model more closely resembled those in the *Vr-*PPase template (PDB: 5GPJ).

### Coarse-grained molecular dynamics (CG-MD) simulations

All CG-MD simulations used the MARTINI 2.2 forcefield [51] and GROMACS 5.0.X (www.gromacs.org) [52]. The crystal structures or homology model were converted into coarse-grained (CG) models and centred independently in 16 x 16.5 x 16 nm^3^ simulation boxes. An elastic network was applied to the backbone atoms within 0.7 nm with a force constraint of 1000 kJmol^-1^nm^2^. This network maintained the secondary and tertiary structure of the proteins but allowed flexible regions, such as loops, to move (**S8 and S9 Figs**). The CG proteins were energy minimised using the steepest descent algorithm embedded in GROMACS. Lipid bilayers were assembled around the proteins and the simulation boxes solvated by random placement of water and NaCl (150 mM) using insane protocols [53]. For *Tm-*PPase and *Cp-*PPase, the bilayers were made up of the palmitoyl-oleoyl phospholipid POPE in combination with anionic POPA, POPG or POPS (**Table 1**). *Vr-*PPase was simulated in a bilayer composed of its native lipid constituents (cholesterol (29%), POPC (25%), POPE (17%), ceramide hexoside (17%), PIP_2_ (6%), POPG (3%), POPS (2%), POPA (1%)) [29]. All systems were generated through this method as shown in Table 2. The complete lipidated and solvated systems were again energy minimised and equilibrated for 2 ns at 323 K, where the backbone particles were restrained. 5 independent repeat production simulations with different initial starting velocities were performed for 5 μs each, which was adequate to reach system convergence (**S11 Fig**). The temperature and pressure of the systems were maintained by a V-rescale thermostat (323 K) and a Parrinello-Rahman barostat (1 bar). The integration timestep was 20 fs and frames were written to the trajectory file every 400 ps, all of which were used in further analysis. The temperature of the systems was initially chosen to accommodate the thermophilic *Tm-*PPase and be above the lipid transition temperatures of all bilayer components, but 310 K was also investigated for *Vr-*PPase (**S7 Fig**).

### CG-AT conversion

A serial multiscale approach was used, in which representative final frames of the selected CG simulations were converted to atomistic (AT) resolutions (“backmapping”). This conversion took place using the Backward protocol as described previously [54]. From each backmapped system, three replicates were generated with randomised starting velocities. These AT simulations were carried out using the CHARMM-36 forcefield [55], a Nosé-Hoover thermostat (323 K), Parrinello-Rahman barostat (1 bar) and 2 fs timestep.

### Analysis

All analyses were performed using GROMACS (gmx mindist, gmx densmap, gmx rms, gmx rmsf), VMD (electrostatic profile, alignments) [56], PyMol (alignments and electron density inspection) [57], PyLipID [28] and locally written scripts. For the contacts between lipid and protein, a locally written script calculated the interactions between each residue and the lipid head groups. A cut-off of 5.5 Å or 4 Å was defined for CG or AT simulations, respectively. For normalisation, all replicates were concatenated, and the number of contacts normalised to the total number of frames and number of that lipid species in the bilayer. The electrostatic profiles of protein structures were calculated by preparing the structure *via* PDB2PQR [58] and then processed using the APBS plugin for VMD [59]. The membrane heightmaps were calculated as in [60] (https://github.com/jiehanchong/membrane-depth-analysis).

## Supporting information

S1 Fig The Lipid Interactions with *Tm-*PPase. Normalised number of contacts between the lipids and *Tm*-PPase in the coarse-grained systems **A)** TmPA40, **B)** TmPG40, **C)** TmPS40, **D)** TmMix10, **E)** TmPA20_DSM **F)** TmPG20_DSM **G)** TmPS20_DSM. Density maps depicting the average density of phosphate particles of the anionic lipids in the **H)** TmPA40 **I)** TmPG40, **J)** TmPS40, **K-M)** POPA, POPG and POPS, respectively, from the TmMix10 system, and **N)** TmPA20_DSM **O)** TmPG20_DSM **P)** TmPS20_DSM systems. **Q-U)** Density maps depicting the average density of phosphate particles of the POPE in the TmPA20 system, with each of the 5 μs simulation repeats shown separately. The number of POPA contacts with the **V)** interfacial and **W)** distal interaction sites over time. Reduced density in the corners of the plots is due to fitting of the trajectory around the protein.
(TIFF)

S2 Fig The Lipid Interactions with *Vr-*PPase. Normalised number of contacts between *Vr*-PPase during coarse-grained simulations and the bilayer representing a realistic tonoplast membrane comprised of **A)** Cholesterol, **B)** POPC, **C)** POPE, **D)** DPCE, **E)** PIP_2_, **F)**, POPG, **G)** POPS and **H)** POPA. Density maps depicting the average density of **I)** cholesterol, or the phosphate particles of **J)** POPC, **K)** POPE, **L)** DPCE, **M)** PIP_2_, **N)** POPG, **O)** POPS and **P)** POPA. The number of contacts between anionic phosphate beads and the **Q-R)** distal interaction site over 5 or 20 μs, respectively, and the **S-T)** interfacial interaction site over 5 or 20 μs, respectively. **U-B’)** The lipid density as in J-P over 20 μs of simulation time.
(TIFF)

S3 Fig The Lipid Interactions with *Cp-*PPase. Normalised number of contacts between the lipids and *Cp*-PPase during coarse-grained simulations in the systems **A)** CpPA20, **B)** CpPG20, **C)** CpPS20, **D)** CpPA20_DSM, **E)** CpPG20_DSM and **F)** CpPS20_DSM. Density maps depicting the average density of phosphate particles of the anionic lipids in the systems **G)** CpPA20, **H)** CpPG20, **I)** CpPS20, **J-L)** POPA, POPG and POPS in CpMix10, **M)** CpPA20_DSM, **N)** CpPG20_DSM and **O)** CpPS20_DSM. **P-Q)** The number of contacts over time between the distal and interfacial interaction site and POPA in the CpPA20 systems, respectively.
(TIFF)

S4 Fig The Lipid Residence Time at *Tm-*PPase Residues. The lipid residence plots and details generated through PyLipID for the residues of the **A-I)** distal interaction site and the **J-M)** interfacial interaction site in the TmPA20 systems.
(TIFF)

S5 Fig The Lipid Residence Time at *Vr-*PPase Residues. The lipid residence plots and details generated through PyLipID for the residues of the **A-I)** distal interaction site and the **J-M)** interfacial interaction site in the VrTonoplast systems.
(TIFF)

S6 Fig The Lipid Residence Time at *Cp-*PPase Residues. The lipid residence plots and details generated through PyLipID for the residues of the **A-I)** distal interaction site and the **J-M)** interfacial interaction site in the CpPA20 systems.
(TIFF)

S7 Fig The Lipid Contacts with *Vr*-PPase at 310K. The lipid interactions with Vr-PPase at 310 K over 5 μs represented through A) normalised lipid contacts, B-C) the number of contacts over time with the interfacial and distal interaction sites, respectively. The lipid density of D) cholesterol, E) POPC, F) POPE, G) DPCE, H) PIP_2_, I) POPG, J) POPS and K) POPA.
(TIFF)

S8 Fig The Dynamics of Atomistic *Tm-*PPase and *Vr-*PPase. The RMSD/C_α_ of the whole protein (black) or the helical part of the protein (blue) of *Tm*-PPase during 250 ns of atomistic resolution simulation in systems **A)** TmPA20, **B)** TmPG20 and **C)** TmPS20, and **D-F)** RMSF/C_α_ of *Tm*-PPase in the same systems. **G-L)** The RMSD/C_α_ of the whole protein (black) or the helical part of the protein (blue) and RMSFC_α_ of the corresponding double interfacial site mutated version of *Tm-*PPase. **M)** The RMSD/C_α_ and **N)** RMSF/C_α_ of *Vr-*PPase during 250 ns of atomistic simulation in the tonoplast bilayer model. **O-P)** the RMSD and RMSF of the CG TmPA20 and **Q-R)** VrTonoplast systems over 5 μs.
(TIFF)

S9 Fig The Dynamics of Homology-Modelled Atomistic *Cp-*PPase. The RMSD/C_α_ of homology-modelled *Cp*-PPase during 250 ns of atomistic resolution simulation in systems **A)** CpPA20, **B)** CpPG20 and **C)** CpPS20, and **D-F)** RMSF/C_α_ of *Cp*-PPase in the same systems. **G-L)** The RMSD/C_α_ and RMSFC_α_ of the corresponding double interfacial site mutated version of *Cp-*PPase. **M-N)** The RMSD and RMSF of the CG CpPA20 system over 5 μs.
(TIFF)

S10 Fig The Homology Models of *Cp-*PPase. The electrostatic profile and Z-Scores of the homology models of *Cp-*PPase generated by **A-B)** Robetta and **C-D)** AlphaFold2. The comparison of **E)** Robetta (cyan) model and the **F)** Alphafold2 model (purple) with the *Vr-*PPase structure (light pink) (PDB: 5GPJ).
(TIFF)

S11 Fig Convergence analysis of *Tm-*PPase 5 μs simulations. The normalised number of contacts between POPA (black) and POPE (red) and *Tm*-PPase in the CG TmPA20 system following **A)** 0–0.1 μs, **B)** 0–0.25 μs, **C)** 0–0.5 μs, **D)** 0–1 μs, **E)** 0–2 μs, **F)** 0–3 μs, **G)** 0–4 μs, **H)** 0–5 μs **I)** 2–5 μs of simulation time. The box size in X of the **J)** TmPA20, **K)** VrTonoplast and **L)** CpPA20 systems during simulation, and the membrane height maps of the **M)** TmPA20, **N)** VrTonoplast and **O)** CpPA20 systems following averaging for all repeats.
(TIFF)

S12 Fig Membrane leaflet dependent density analysis. The density maps depicting the average density of phosphate particles of POPE in the **A)** luminal and **B)** cytoplasmic leaflet and the POPA in the **C)** luminal and **D)** cytoplasmic leaflet of the TmPA20 system. The average density of phosphate particles of POPE in the **E)** luminal and **F)** cytoplasmic leaflet and the POPG in the **G)** luminal and **H)** cytoplasmic leaflet of the TmPG20 system. The average density of phosphate particles of POPE in the **I)** luminal and **J)** cytoplasmic leaflet and the POPS in the **K)** luminal and **L)** cytoplasmic leaflet of the TmPS20 system.
(TIFF)

S13 Fig Normalised lipid contacts in backmapped systems. The normalised lipid contacts between the bilayer and lipids in the **A)** TmPA20, **B)** TmPG20, **C)** TmPS20, **D)** CpPA20, **E)** CpPG20, **F)** CpPS20 and **G-N)** VrTonoplast systems following 250 ns of simulations.
(TIFF)
