# Supplementary figures and images for: mPPases create a conserved anionic membrane fingerprint as identified via multi-scale simulations
Source: PLoS Comput Biol. 2022 Oct 3;18(10):e1010578. doi: 10.1371/journal.pcbi.1010578 (PMC9560603; doi:10.1371/journal.pcbi.1010578)

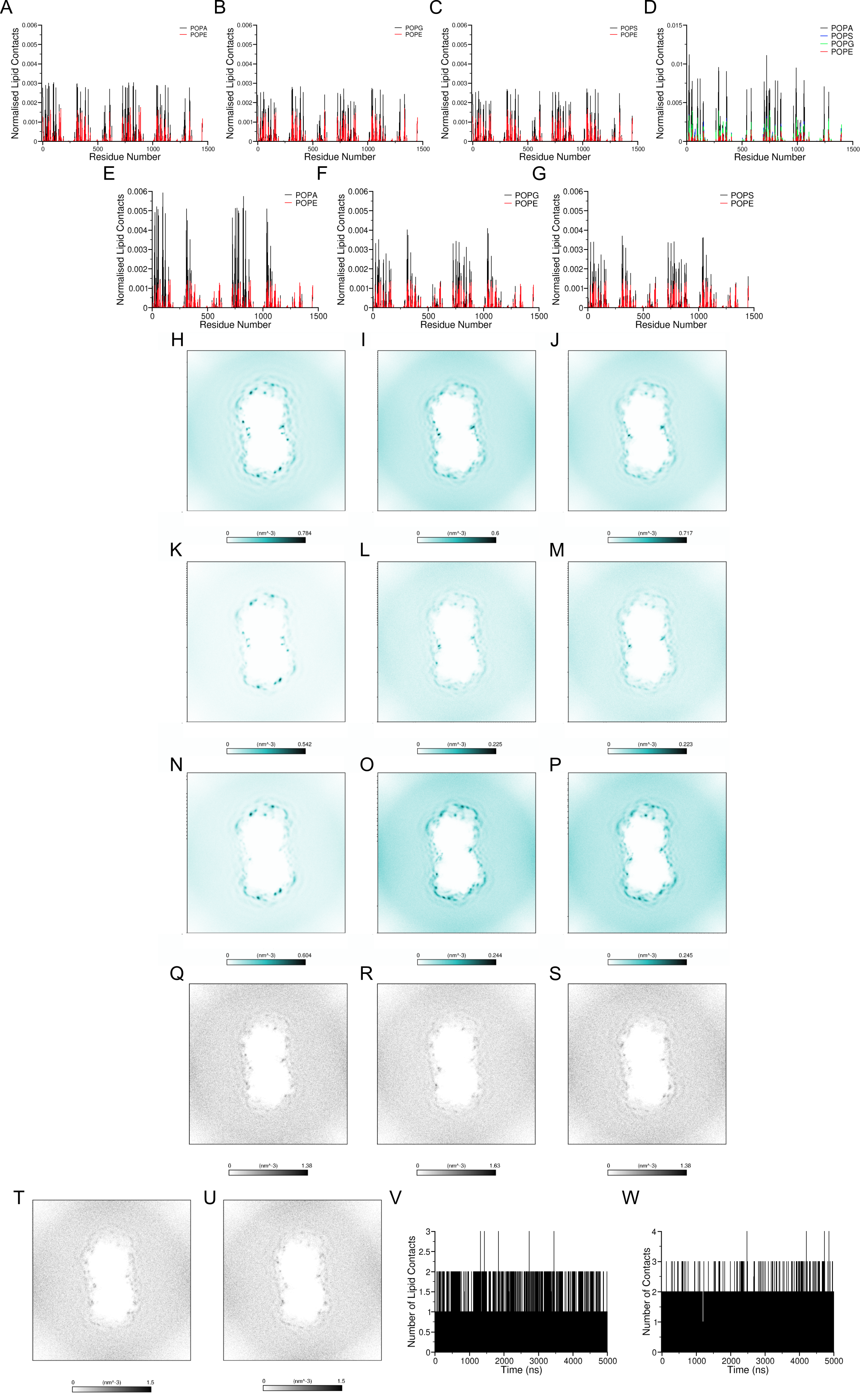

Supplement: S1 Fig — Normalised number of contacts between the lipids and Tm-PPase in the coarse-grained systems A) TmPA40, B) TmPG40, C) TmPS40, D) TmMix10, E) TmPA20_DSM F) TmPG20_DSM G) TmPS20_DSM. Density maps depicting the average density of phosphate particles of the anionic lipids in the H) TmPA40 I) TmPG40, J) TmPS40, K-M) POPA, POPG and POPS, respectively, from the TmMix10 system, and N) TmPA20_DSM O) TmPG20_DSM P) TmPS20_DSM systems. Q-U) Density maps depicting the average density of phosphate particles of the POPE in the TmPA20 system, with each of the 5 μs simulation repeats shown separately. The number of POPA contacts with the V) interfacial and W) distal interaction sites over time. Reduced density in the corners of the plots is due to fitting of the trajectory around the protein. (TIFF) [file pcbi.1010578.s001.tiff]

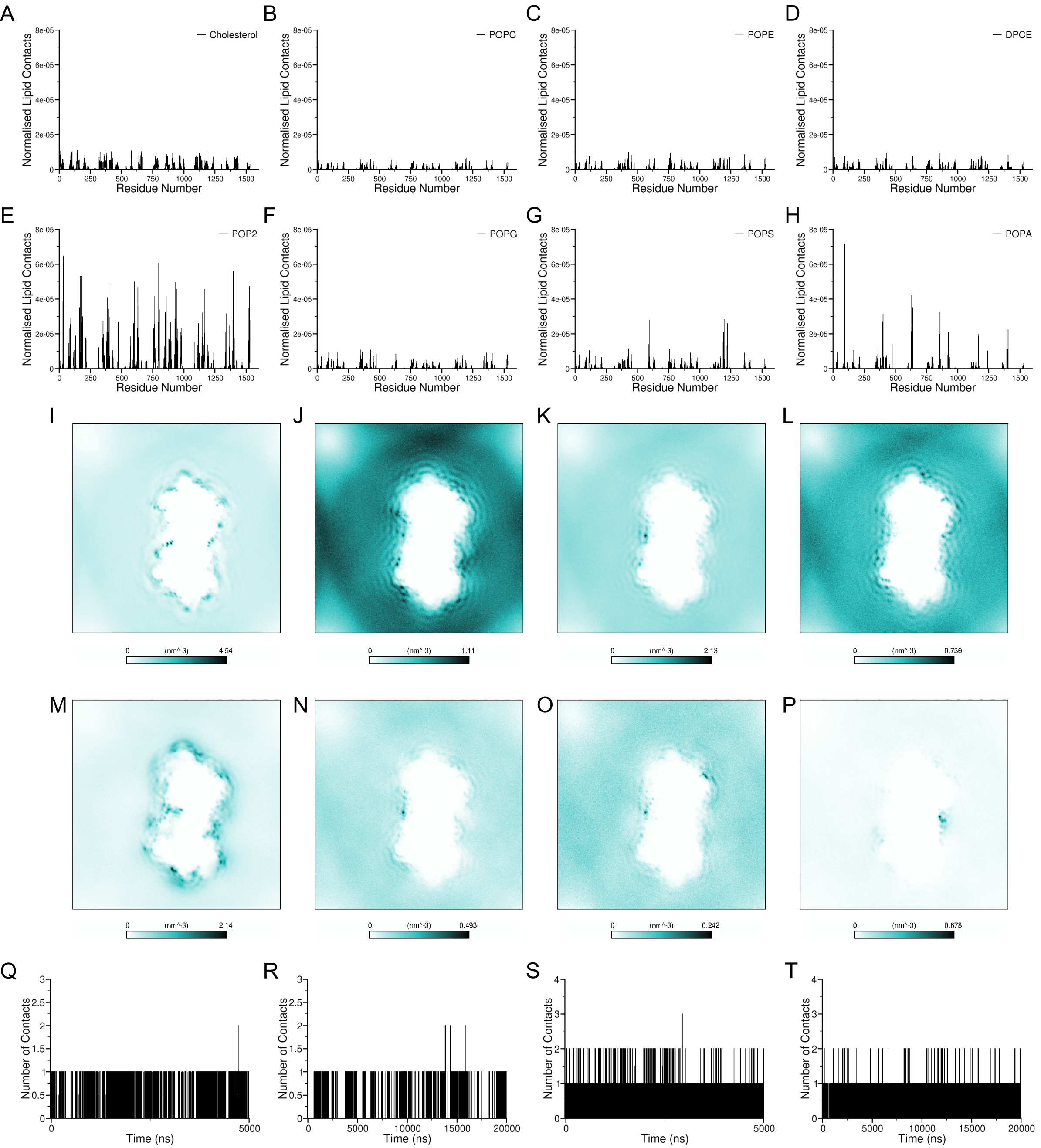

Supplement: S2 Fig — Normalised number of contacts between Vr-PPase during coarse-grained simulations and the bilayer representing a realistic tonoplast membrane comprised of A) Cholesterol, B) POPC, C) POPE, D) DPCE, E) PIP2, F), POPG, G) POPS and H) POPA. Density maps depicting the average density of I) cholesterol, or the phosphate particles of J) POPC, K) POPE, L) DPCE, M) PIP2, N) POPG, O) POPS and P) POPA. The number of contacts between anionic phosphate beads and the Q-R) distal interaction site over 5 or 20 μs, respectively, and the S-T) interfacial interaction site over 5 or 20 μs, respectively. U-B’) The lipid density as in J-P over 20 μs of simulation time. (TIFF) [file pcbi.1010578.s002.tiff]

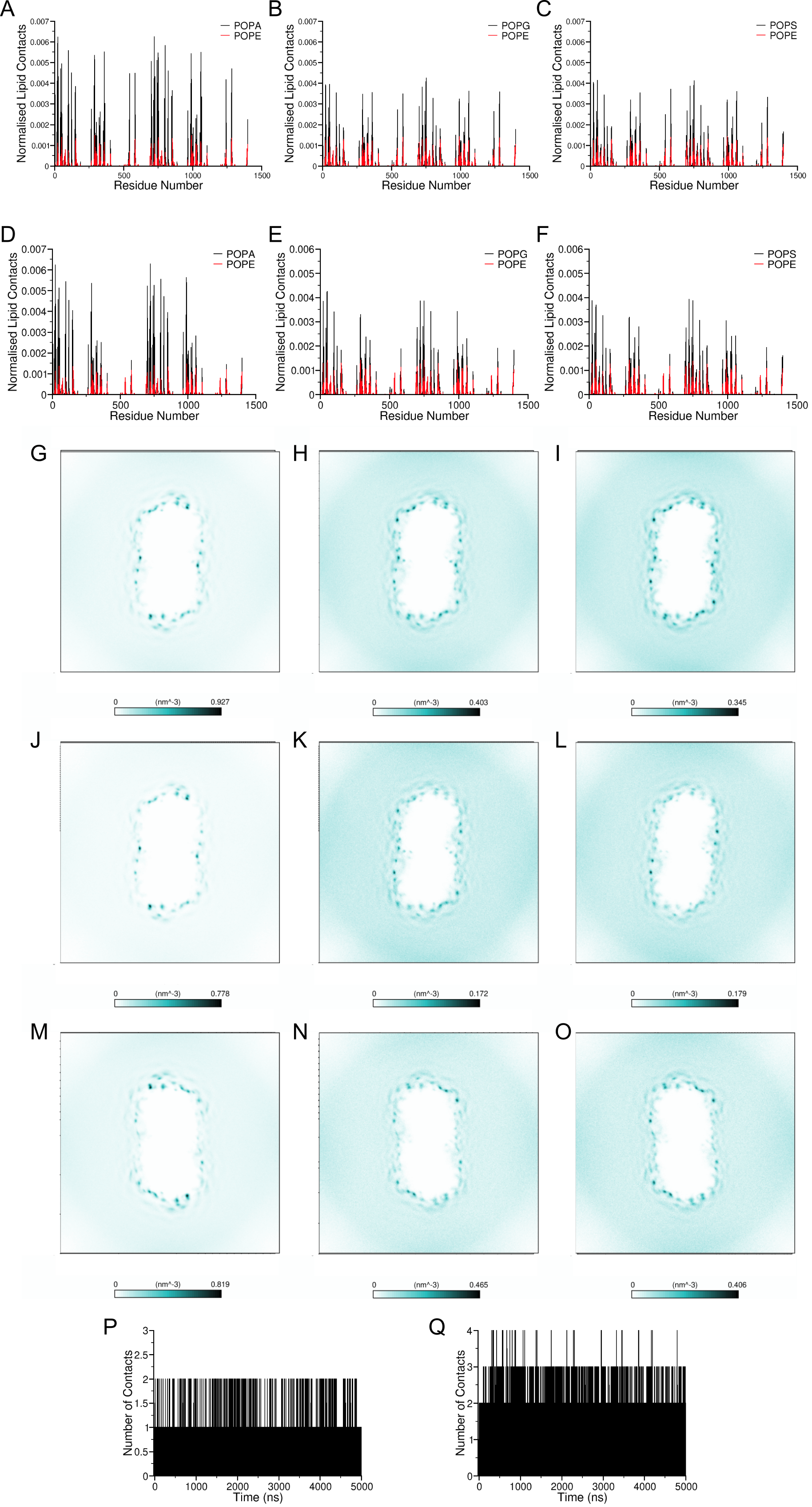

Supplement: S3 Fig — Normalised number of contacts between the lipids and Cp-PPase during coarse-grained simulations in the systems A) CpPA20, B) CpPG20, C) CpPS20, D) CpPA20_DSM, E) CpPG20_DSM and F) CpPS20_DSM. Density maps depicting the average density of phosphate particles of the anionic lipids in the systems G) CpPA20, H) CpPG20, I) CpPS20, J-L) POPA, POPG and POPS in CpMix10, M) CpPA20_DSM, N) CpPG20_DSM and O) CpPS20_DSM. P-Q) The number of contacts over time between the distal and interfacial interaction site and POPA in the CpPA20 systems, respectively. (TIFF) [file pcbi.1010578.s003.tiff]

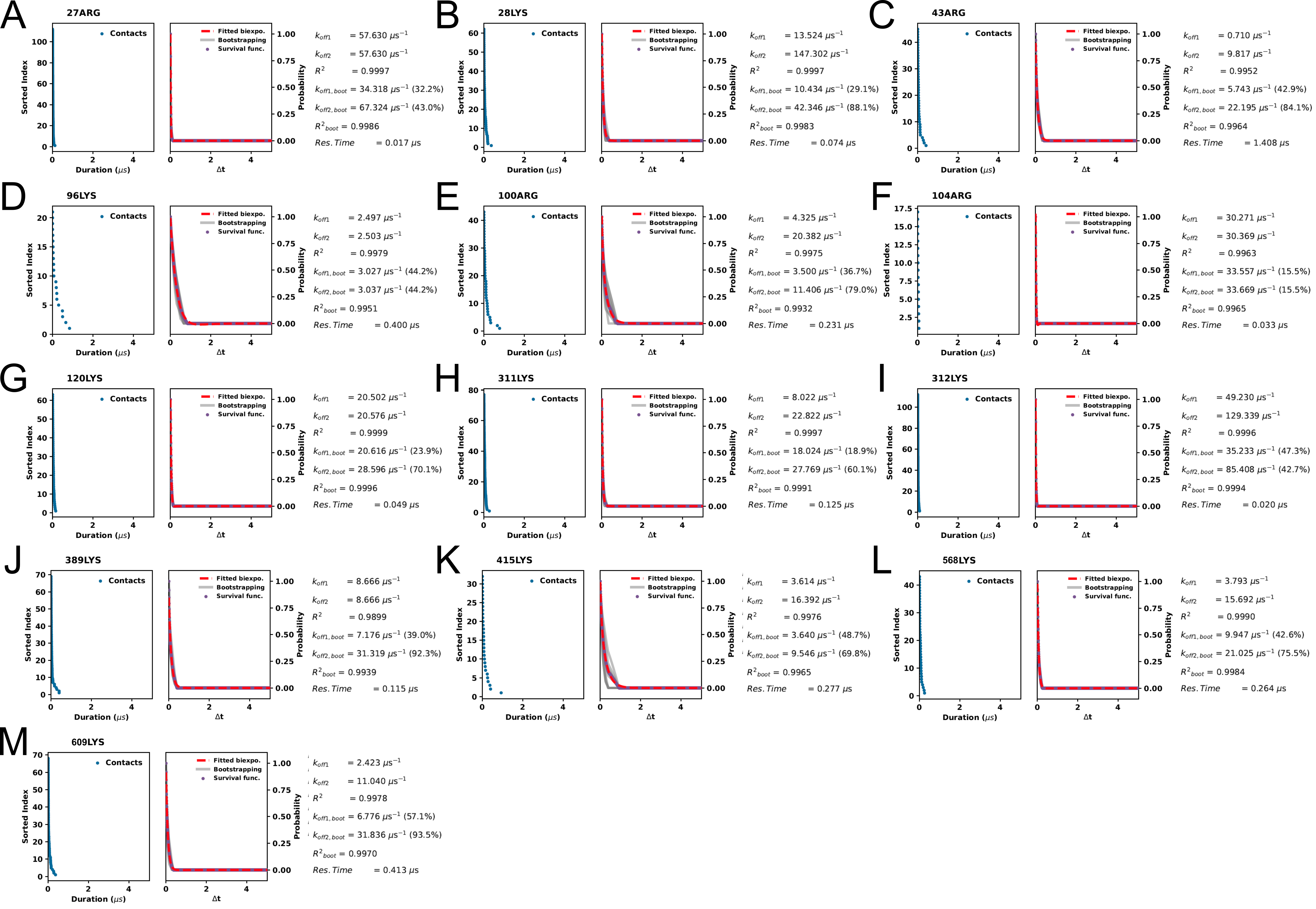

Supplement: S4 Fig — The lipid residence plots and details generated through PyLipID for the residues of the A-I) distal interaction site and the J-M) interfacial interaction site in the TmPA20 systems. (TIFF) [file pcbi.1010578.s004.tiff]

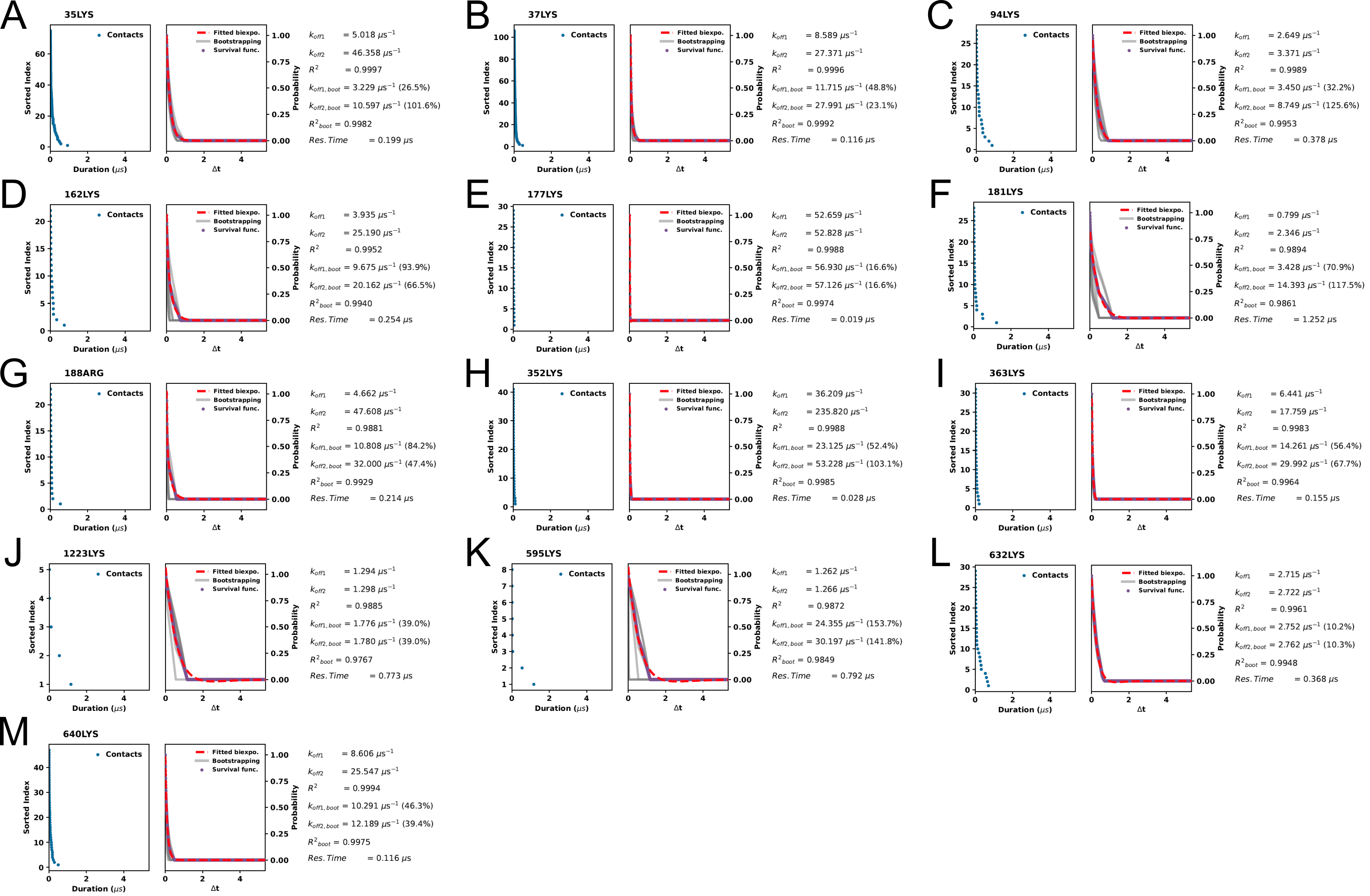

Supplement: S5 Fig — The lipid residence plots and details generated through PyLipID for the residues of the A-I) distal interaction site and the J-M) interfacial interaction site in the VrTonoplast systems. (TIFF) [file pcbi.1010578.s005.tiff]

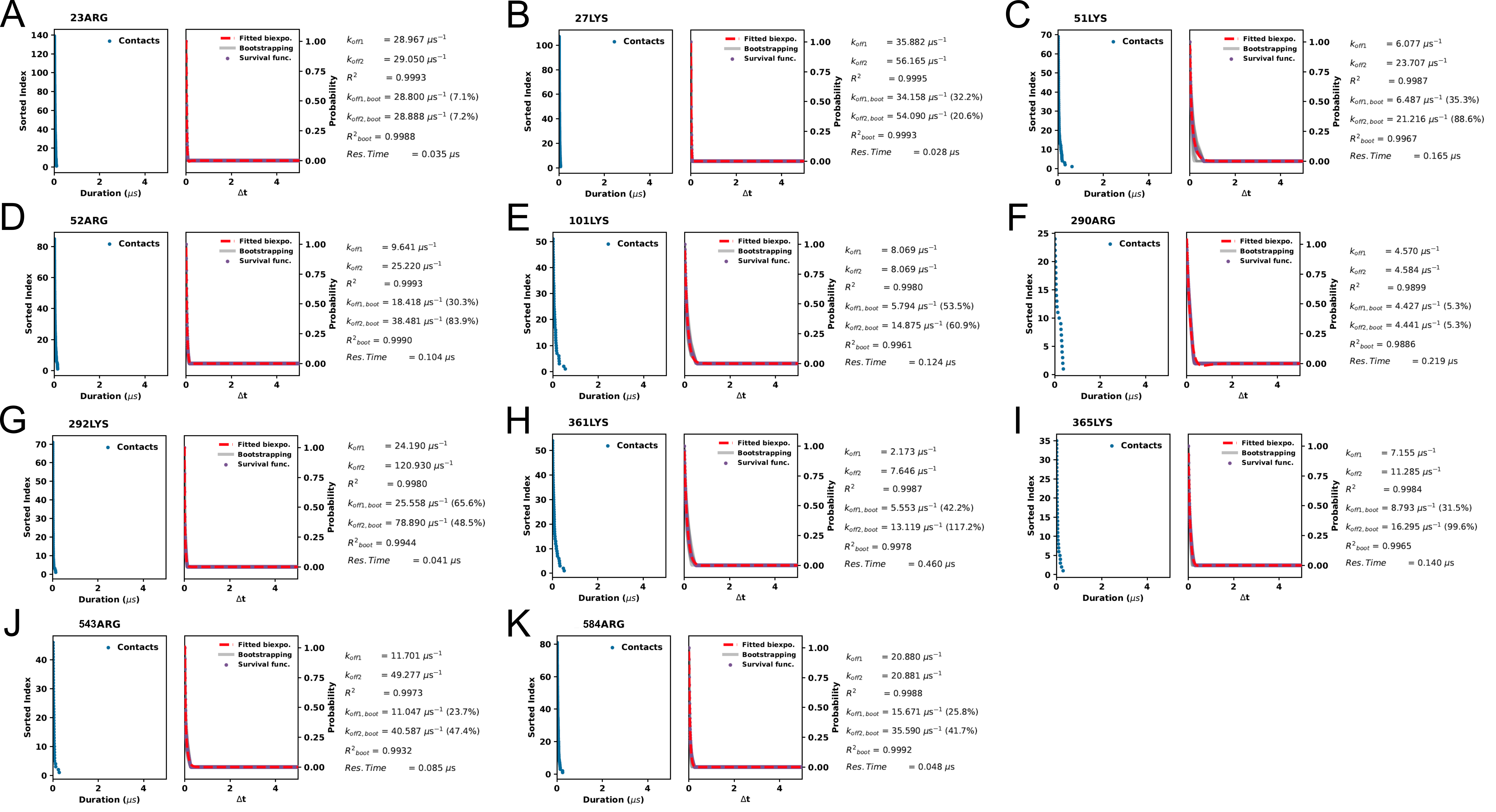

Supplement: S6 Fig — The lipid residence plots and details generated through PyLipID for the residues of the A-I) distal interaction site and the J-M) interfacial interaction site in the CpPA20 systems. (TIFF) [file pcbi.1010578.s006.tiff]

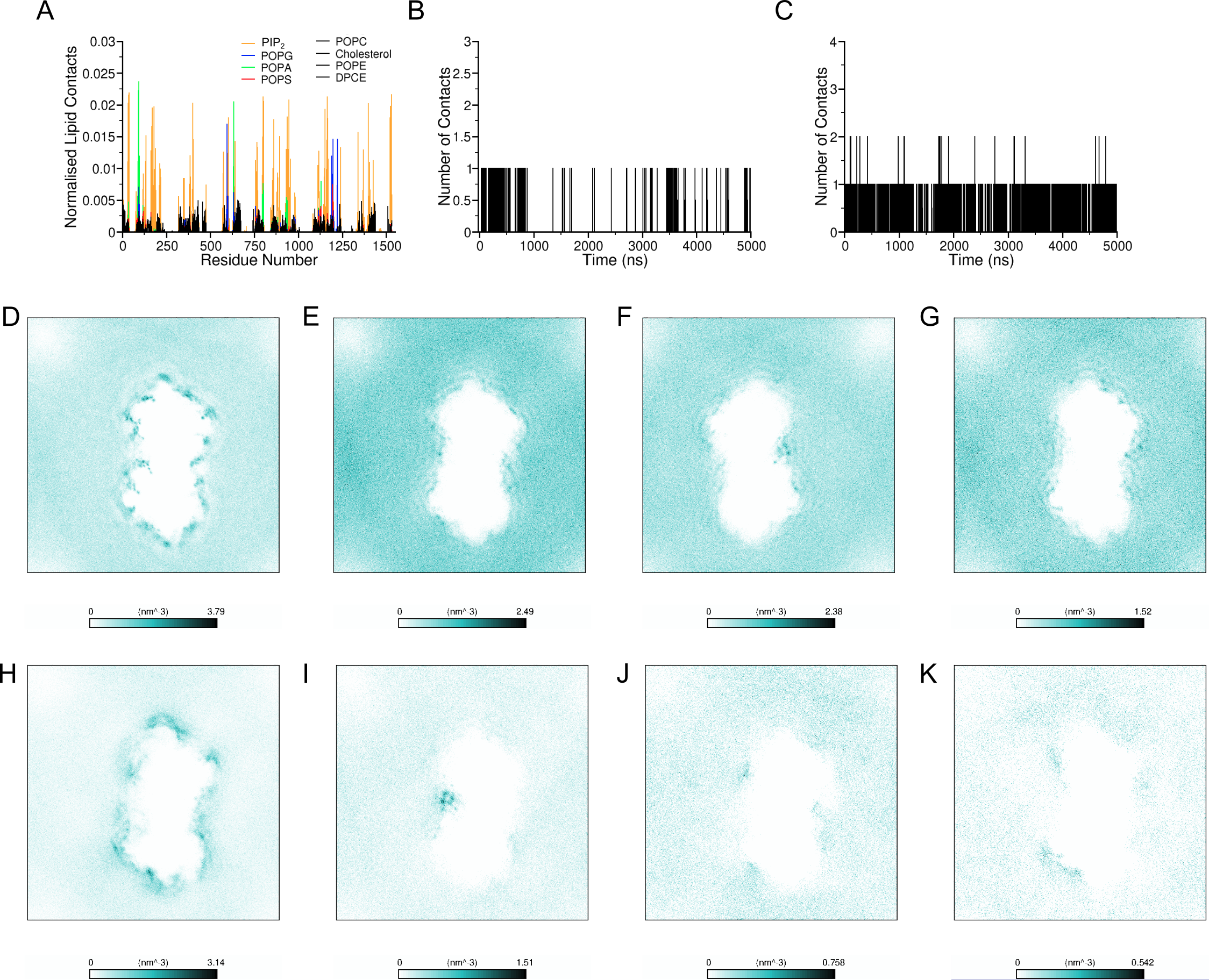

Supplement: S7 Fig — The lipid interactions with Vr-PPase at 310 K over 5 μs represented through A) normalised lipid contacts, B-C) the number of contacts over time with the interfacial and distal interaction sites, respectively. The lipid density of D) cholesterol, E) POPC, F) POPE, G) DPCE, H) PIP2, I) POPG, J) POPS and K) POPA. (TIFF) [file pcbi.1010578.s007.tiff]

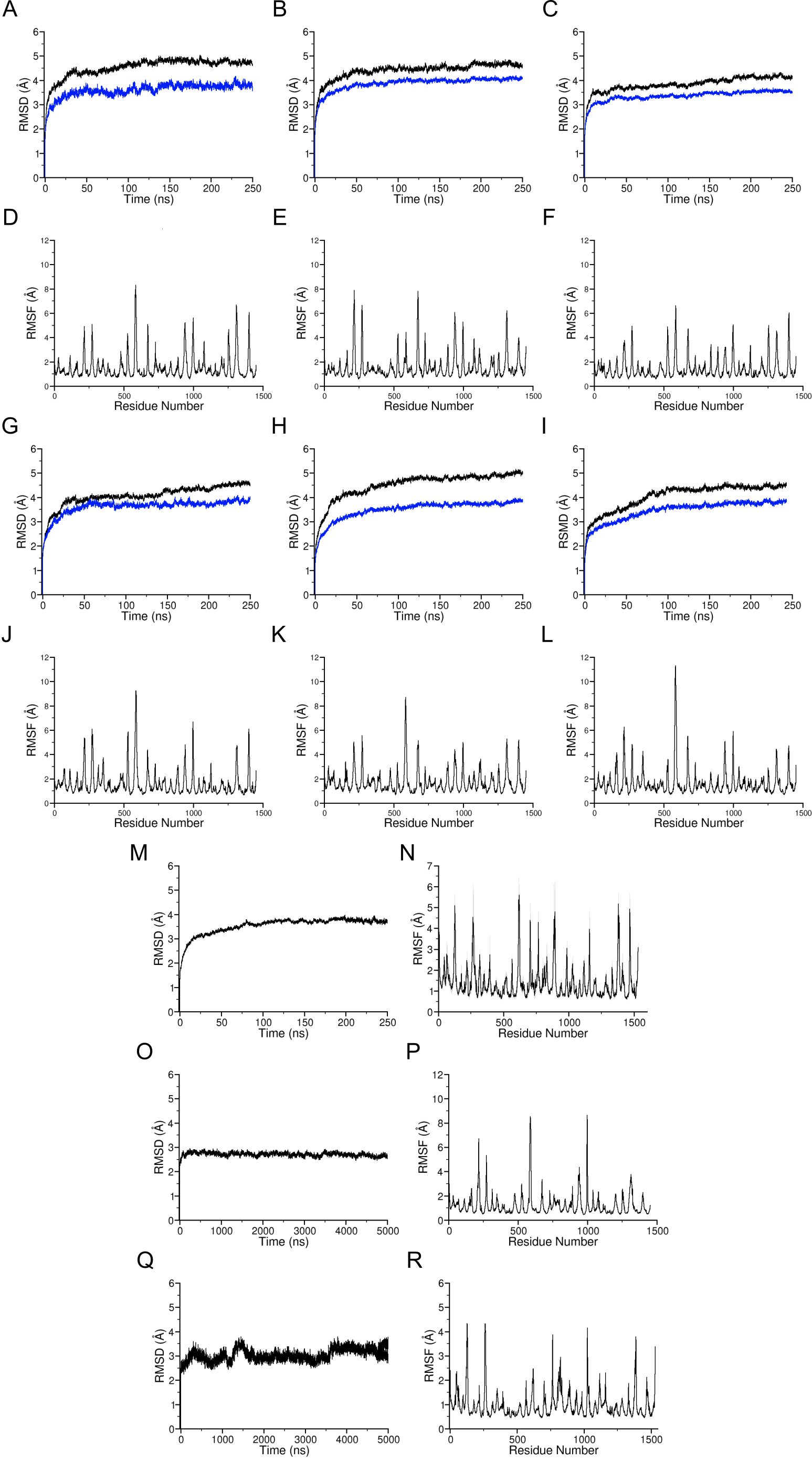

Supplement: S8 Fig — The RMSD/Cα of the whole protein (black) or the helical part of the protein (blue) of Tm-PPase during 250 ns of atomistic resolution simulation in systems A) TmPA20, B) TmPG20 and C) TmPS20, and D-F) RMSF/Cα of Tm-PPase in the same systems. G-L) The RMSD/Cα of the whole protein (black) or the helical part of the protein (blue) and RMSFCα of the corresponding double interfacial site mutated version of Tm-PPase. M) The RMSD/Cα and N) RMSF/Cα of Vr-PPase during 250 ns of atomistic simulation in the tonoplast bilayer model. O-P) the RMSD and RMSF of the CG TmPA20 and Q-R) VrTonoplast systems over 5 μs. (TIFF) [file pcbi.1010578.s008.tiff]

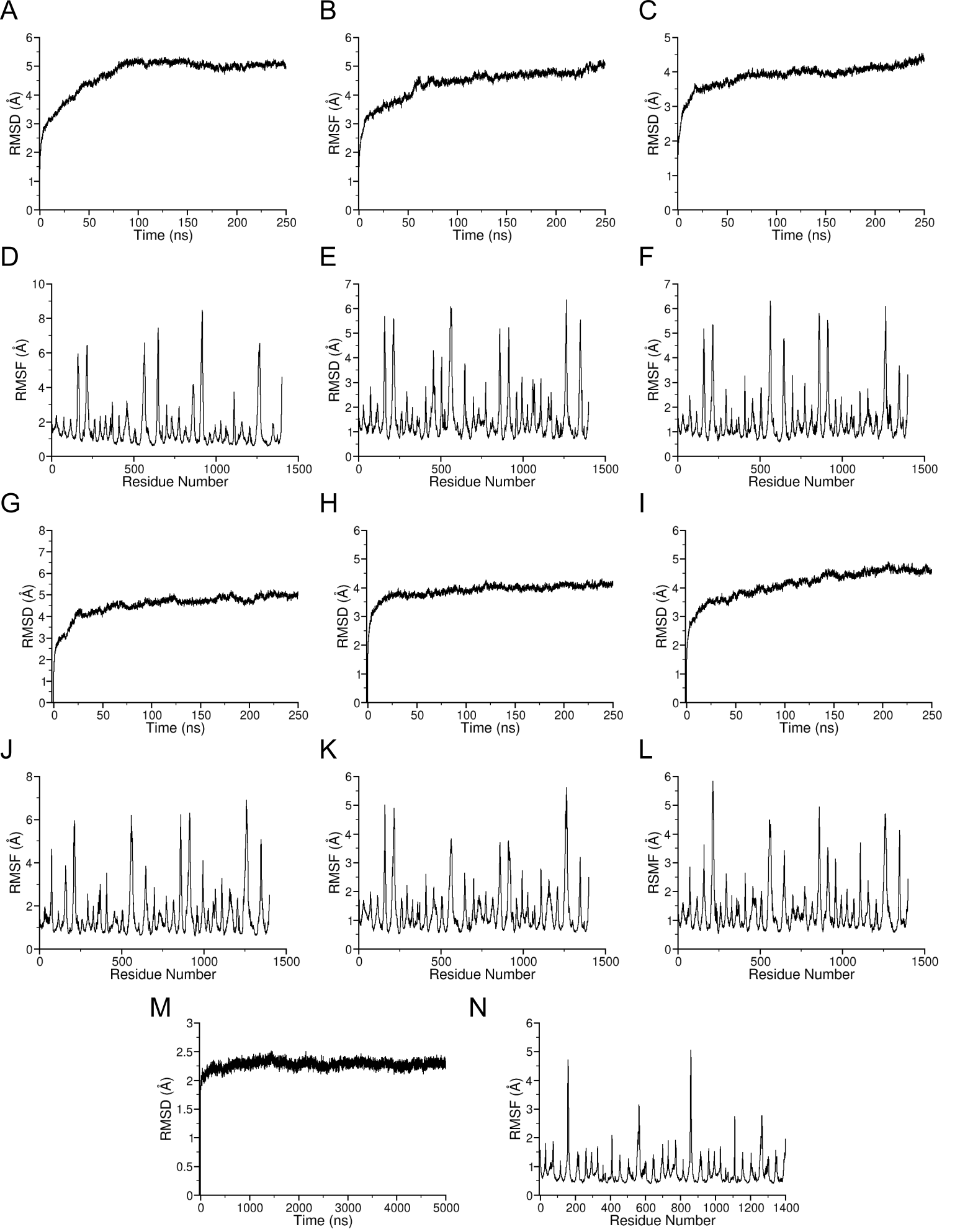

Supplement: S9 Fig — The RMSD/Cα of homology-modelled Cp-PPase during 250 ns of atomistic resolution simulation in systems A) CpPA20, B) CpPG20 and C) CpPS20, and D-F) RMSF/Cα of Cp-PPase in the same systems. G-L) The RMSD/Cα and RMSFCα of the corresponding double interfacial site mutated version of Cp-PPase. M-N) The RMSD and RMSF of the CG CpPA20 system over 5 μs. (TIFF) [file pcbi.1010578.s009.tiff]

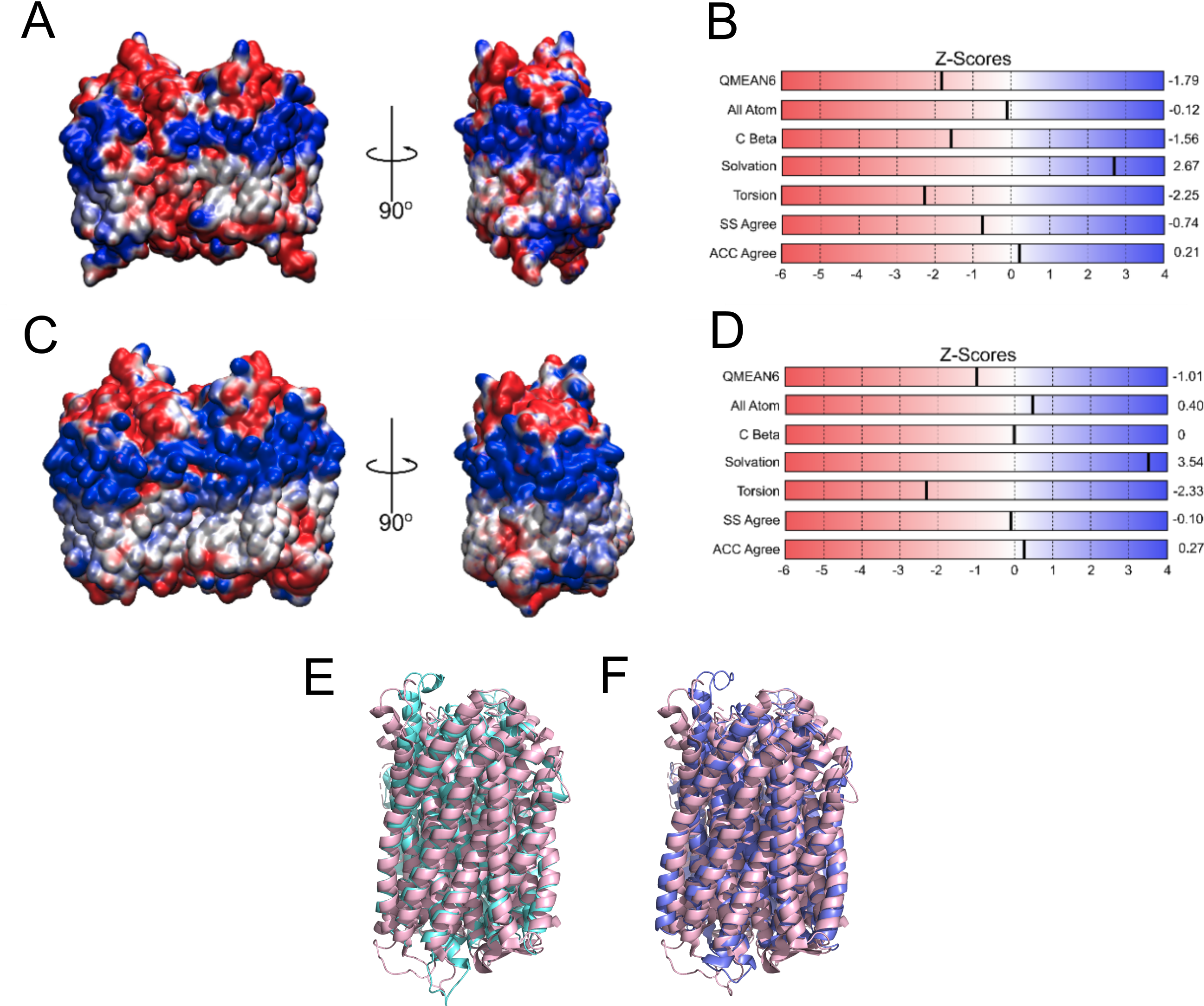

Supplement: S10 Fig — The electrostatic profile and Z-Scores of the homology models of Cp-PPase generated by A-B) Robetta and C-D) AlphaFold2. The comparison of E) Robetta (cyan) model and the F) Alphafold2 model (purple) with the Vr-PPase structure (light pink) (PDB: 5GPJ). (TIFF) [file pcbi.1010578.s010.tiff]

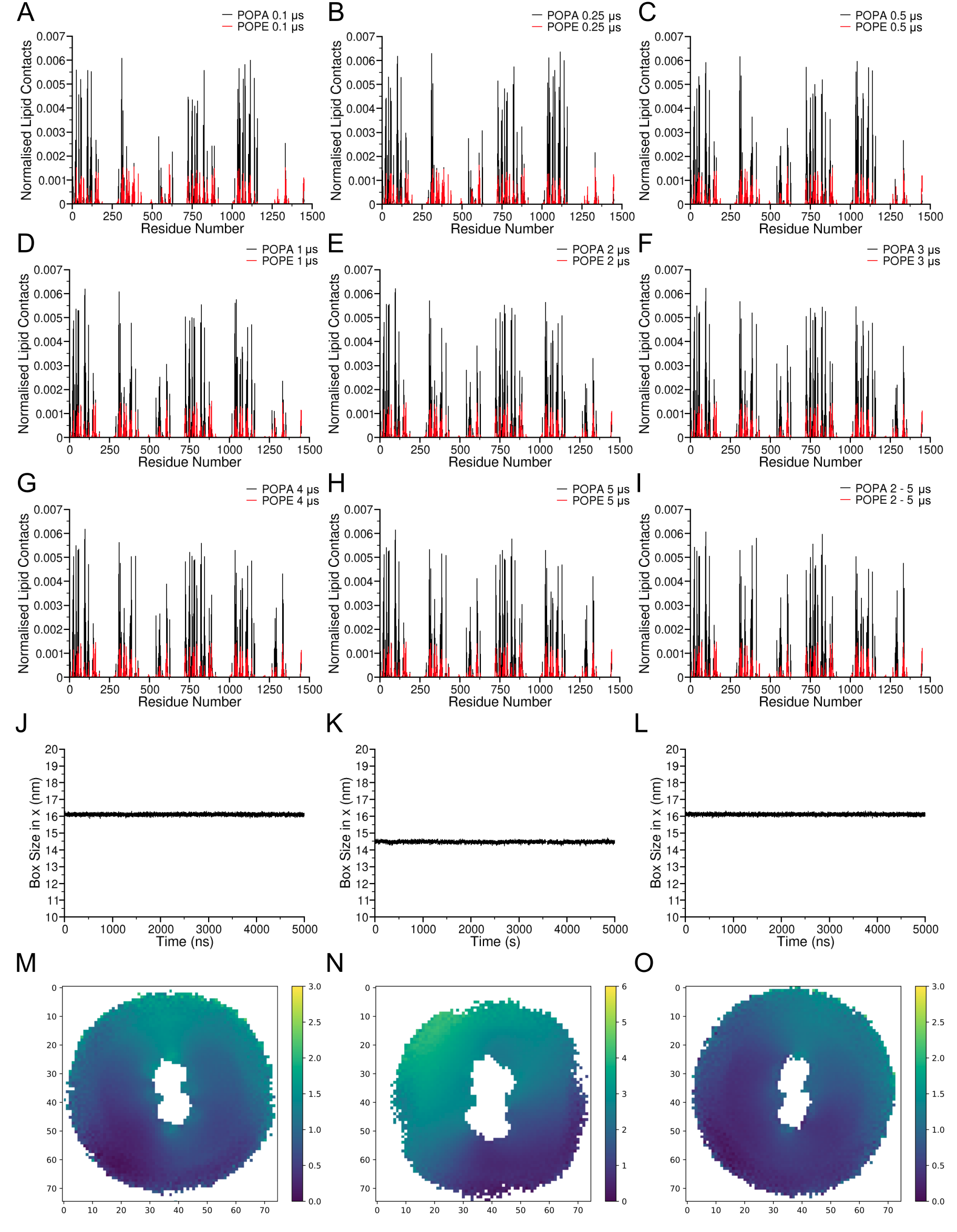

Supplement: S11 Fig — The normalised number of contacts between POPA (black) and POPE (red) and Tm-PPase in the CG TmPA20 system following A) 0–0.1 μs, B) 0–0.25 μs, C) 0–0.5 μs, D) 0–1 μs, E) 0–2 μs, F) 0–3 μs, G) 0–4 μs, H) 0–5 μs I) 2–5 μs of simulation time. The box size in X of the J) TmPA20, K) VrTonoplast and L) CpPA20 systems during simulation, and the membrane height maps of the M) TmPA20, N) VrTonoplast and O) CpPA20 systems following averaging for all repeats. (TIFF) [file pcbi.1010578.s011.tiff]

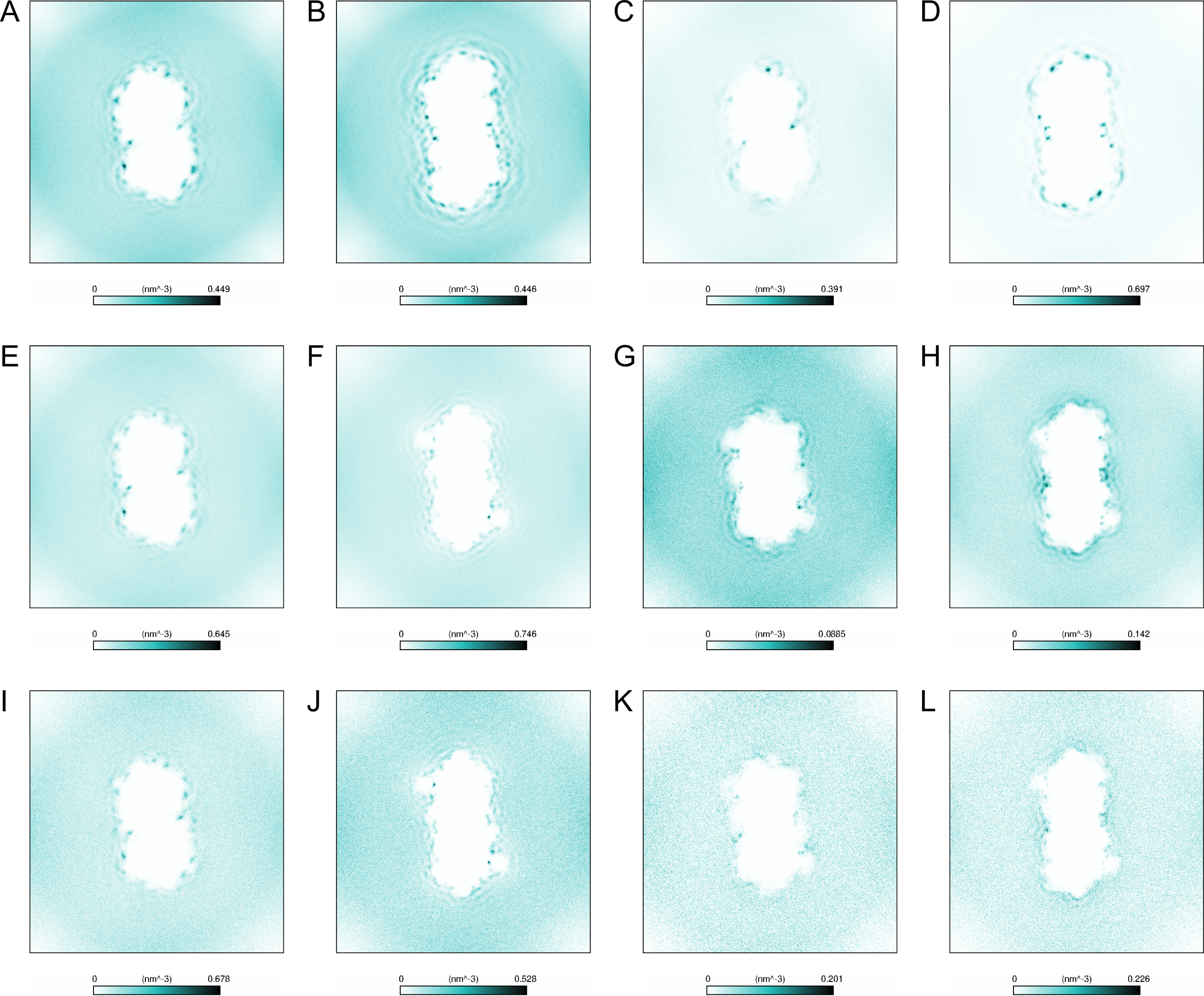

Supplement: S12 Fig — The density maps depicting the average density of phosphate particles of POPE in the A) luminal and B) cytoplasmic leaflet and the POPA in the C) luminal and D) cytoplasmic leaflet of the TmPA20 system. The average density of phosphate particles of POPE in the E) luminal and F) cytoplasmic leaflet and the POPG in the G) luminal and H) cytoplasmic leaflet of the TmPG20 system. The average density of phosphate particles of POPE in the I) luminal and J) cytoplasmic leaflet and the POPS in the K) luminal and L) cytoplasmic leaflet of the TmPS20 system. (TIFF) [file pcbi.1010578.s012.tiff]

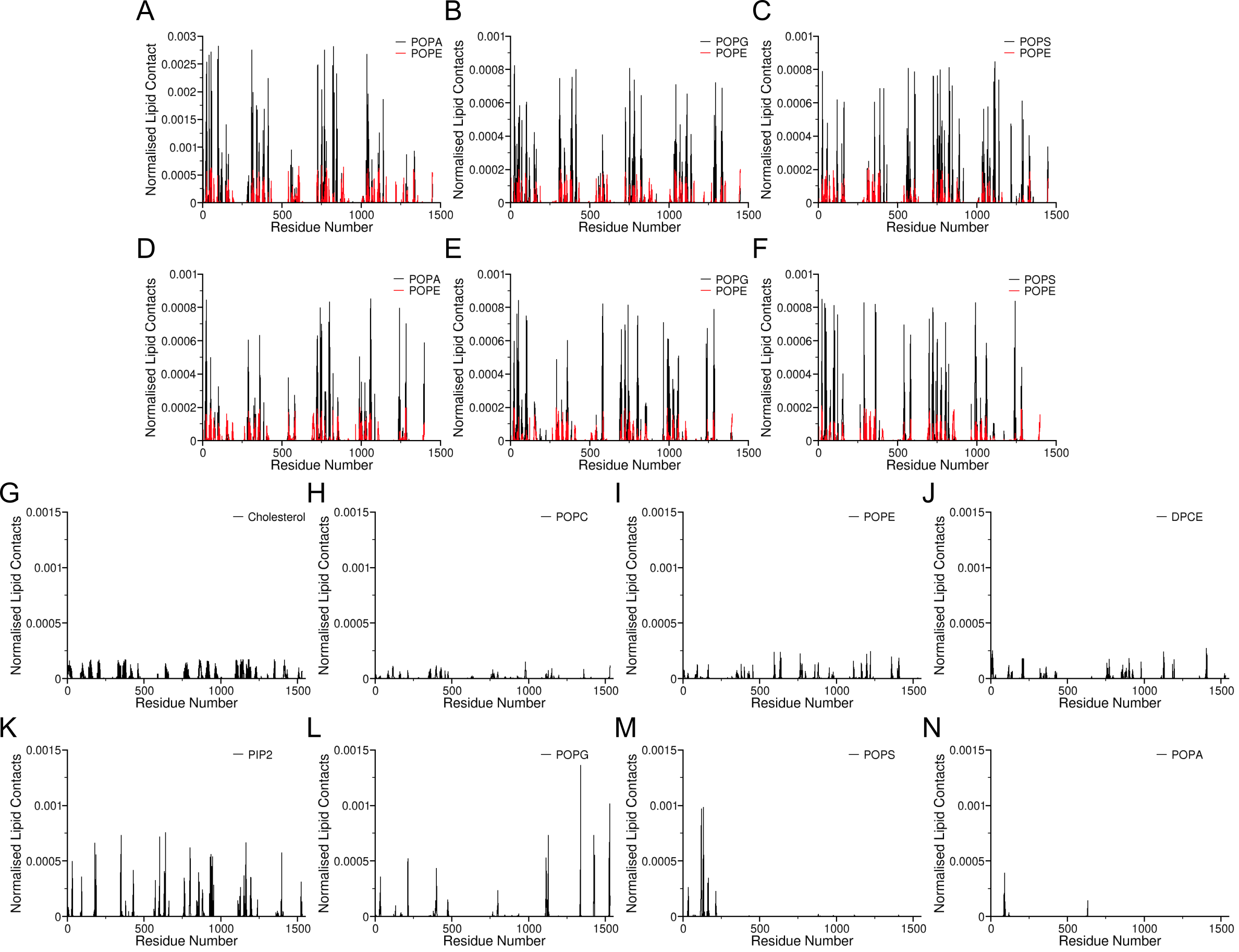

Supplement: S13 Fig — The normalised lipid contacts between the bilayer and lipids in the A) TmPA20, B) TmPG20, C) TmPS20, D) CpPA20, E) CpPG20, F) CpPS20 and G-N) VrTonoplast systems following 250 ns of simulations. (TIFF) [file pcbi.1010578.s013.tiff]
